# Correction: Computational analysis into the potential of azo dyes as a feedstock for actinorhodin biosynthesis in Pseudomonas putida

**DOI:** 10.1371/journal.pone.0304263

**Published:** 2024-05-17

**Authors:** The PLOS One Staff

There are errors in the Funding statement. The correct Funding statement is as follows: The authors thank Lembaga Pengelola Dana Pendidikan (LPDP) of the Republic of Indonesia, Pusat Layanan Pembiayaan Pendidikan (PUSLAPDIK) at the Ministry of Education, Culture, Research and Technology of the Republic of Indonesia, and Maudy Ayunda Foundation’s Project Scholarship for supporting the publication fees for this study. The funders had no role in study design, data collection and analysis, decision to publish, or preparation of the manuscript.

The publisher apologizes for the errors.

## References

[1] Nayyara P, Permana D, Ermawar RA, Fahayana R (2024) Computational analysis into the potential of azo dyes as a feedstock for actinorhodin biosynthesis in Pseudomonas putida. PLoS ONE 19(3): e0299128. 10.1371/journal.pone.0299128 38437212 PMC10911627

