# Supplementary material for: Computational analysis into the potential of azo dyes as a feedstock for actinorhodin biosynthesis in Pseudomonas putida
Source: PLoS One. 2024 Mar 4;19(3):e0299128. doi: 10.1371/journal.pone.0299128 (PMC10911627; doi:10.1371/journal.pone.0299128)
Supplement: S4 File — Shows the production envelopes of strain designs generated by OptKnock, OptGene, and cMCS. (DOCX) [file pone.0299128.s004.docx]

**Supporting Information**

**Computational** **Analysis into the Potential of Azo Dyes as a Feedstock for Actinorhodin Biosynthesis in *Pseudomonas putida***

Parsa Nayyara^1, 2, *^, Dani Permana^3, *^, Riksfardini A. Ermawar^4^, Ratih Fahayana^1^

^1^Sekolah Menengah Atas Negeri (SMAN) 5 Surabaya, Jalan Kusuma Bangsa No. 21, Surabaya 60272, Indonesia

^2^University of British Columbia, Vancouver, British Columbia V6T 1Z4, Canada

^3^Research Center for Genetic Engineering, The National Research and Innovation Agency of the Republic of Indonesia (Badan Riset dan Inovasi Nasional (BRIN)), Kawasan Sains dan Teknologi (KST) Ir. Soekarno, Jalan Raya Jakarta Bogor, KM. 46, Cibinong, Bogor 16911, Indonesia

^4^Research Center for Biomass and Bioproducts, National Research and Innovation Agency, Republic of Indonesia (BRIN), Kawasan Sains dan Teknologi (KST) Ir. Soekarno, Jalan Raya Jakarta Bogor, KM. 46, Cibinong, Bogor 16911, Indonesia

Corresponding Authors:

*E-mail : [nayyara@student.ubc.ca](mailto:nayyara@student.ubc.ca); [dani008@brin.go.id](mailto:dani008@brin.go.id)

**Table of Contents**

**Figures**

Figure 1. Production envelopes of strain designs suggested by OptKnock. Engineering objective/target: MRt1.

Figure 2. Production envelopes of strain designs suggested by OptKnock. Engineering objective/target: ACTt. Carbon source: GLC

Figure 3. Production envelopes of strain designs suggested by OptKnock. Engineering objective/target: ACTt. Carbon source: MR

Figure 4. Production envelopes of strain designs suggested by OptKnock. Engineering objective/target: ACTt. Carbon source: GLC+MR.

Figure 5. Production envelopes of strain designs suggested by cMCS.

**OptKnock Production Envelopes**


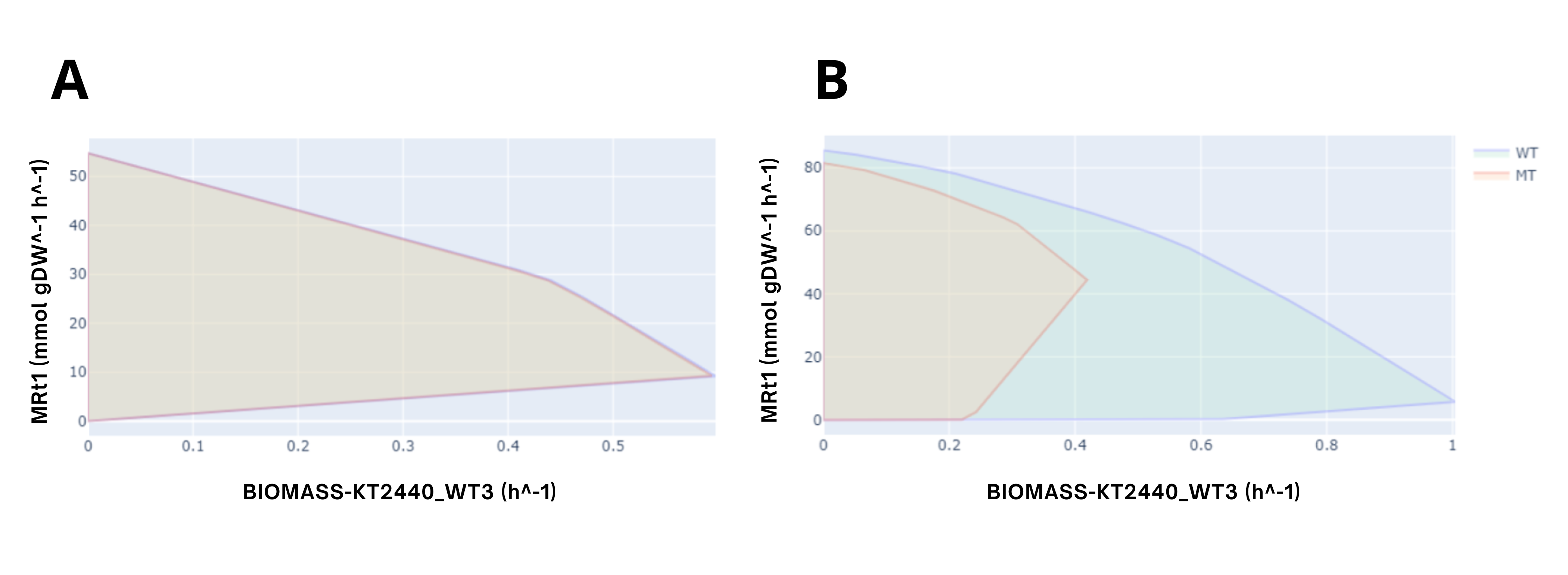


**Figure 1.** Production envelopes of strain designs suggested by OptKnock. Engineering objective/target: MRt1. (A) ACS knockout, carbon source: MR (B) ATPS4rpp knockout, carbon source: GLC + MR. WT: iJN1462c; MT: mutant-type/knocked out strain design


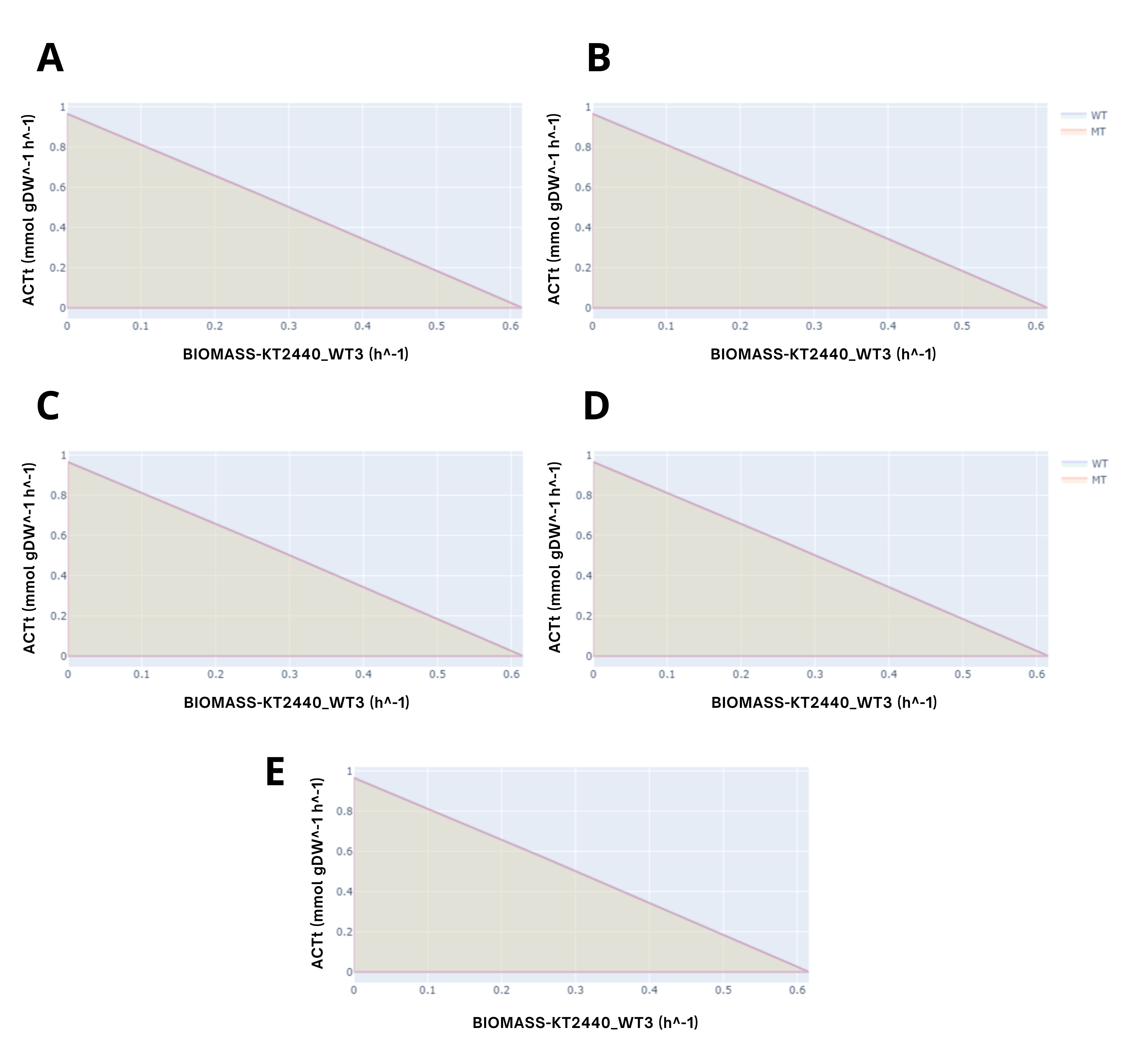


**Figure 2.** Production envelopes of strain designs suggested by OptKnock. Engineering objective/target: ACTt. Carbon source: GLC. (A) FACOAE90 knockout (B) ADK1 knockout (C) KAS14 knockout (D) HBRCH knockout (E) ALGAC5 knockout. WT: iJN1462c; MT: mutant-type/knocked out strain design


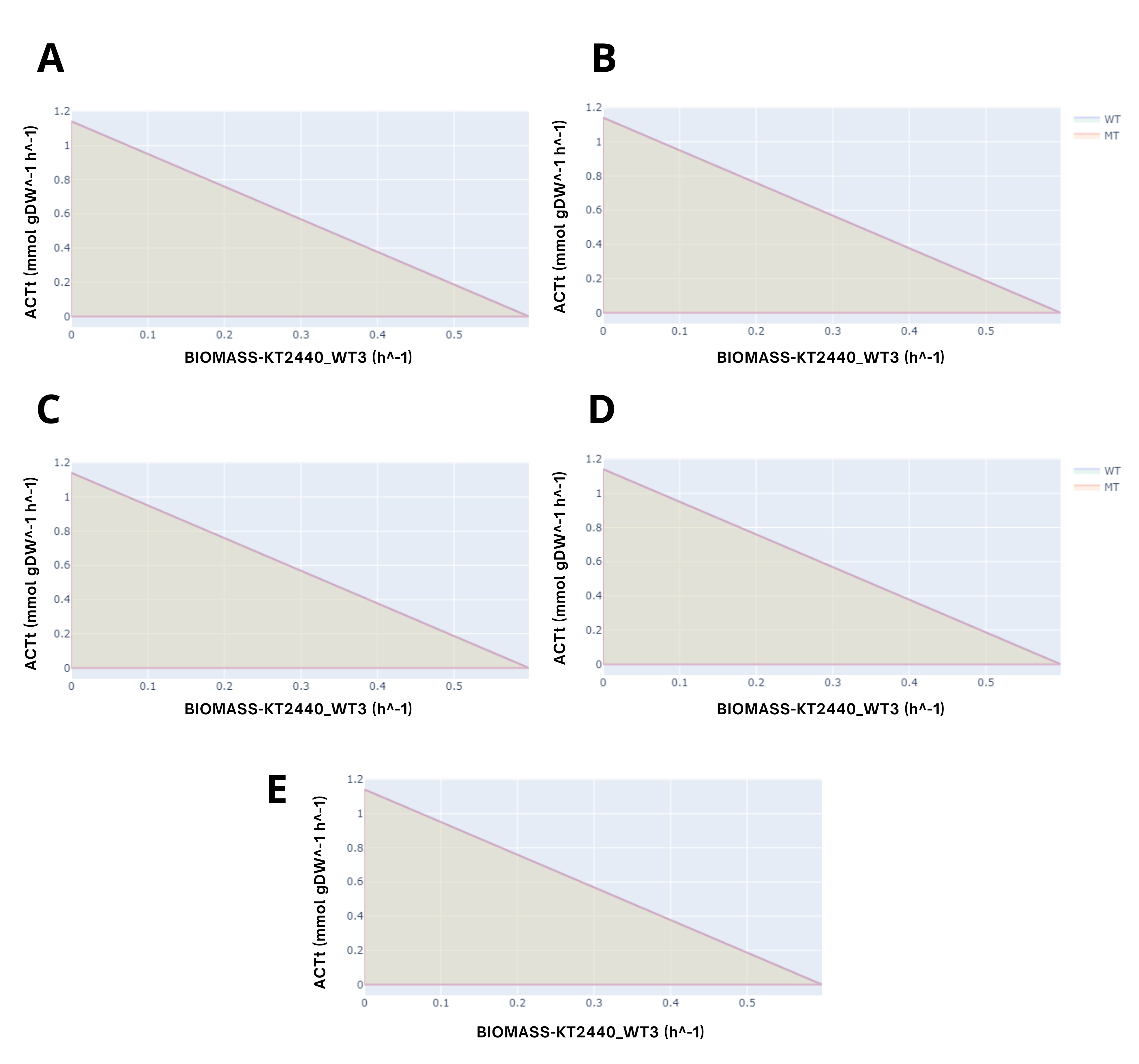


**Figure 3.** Production envelopes of strain designs suggested by OptKnock. Engineering objective/target: ACTt. Carbon source: MR. (A) QUIDH knockout (B) GLXCL knockout (C) AATA knockout (D) TYRTA knockout (E) GGDAPS knockout. WT: iJN1462c; MT: mutant-type/knocked out strain design


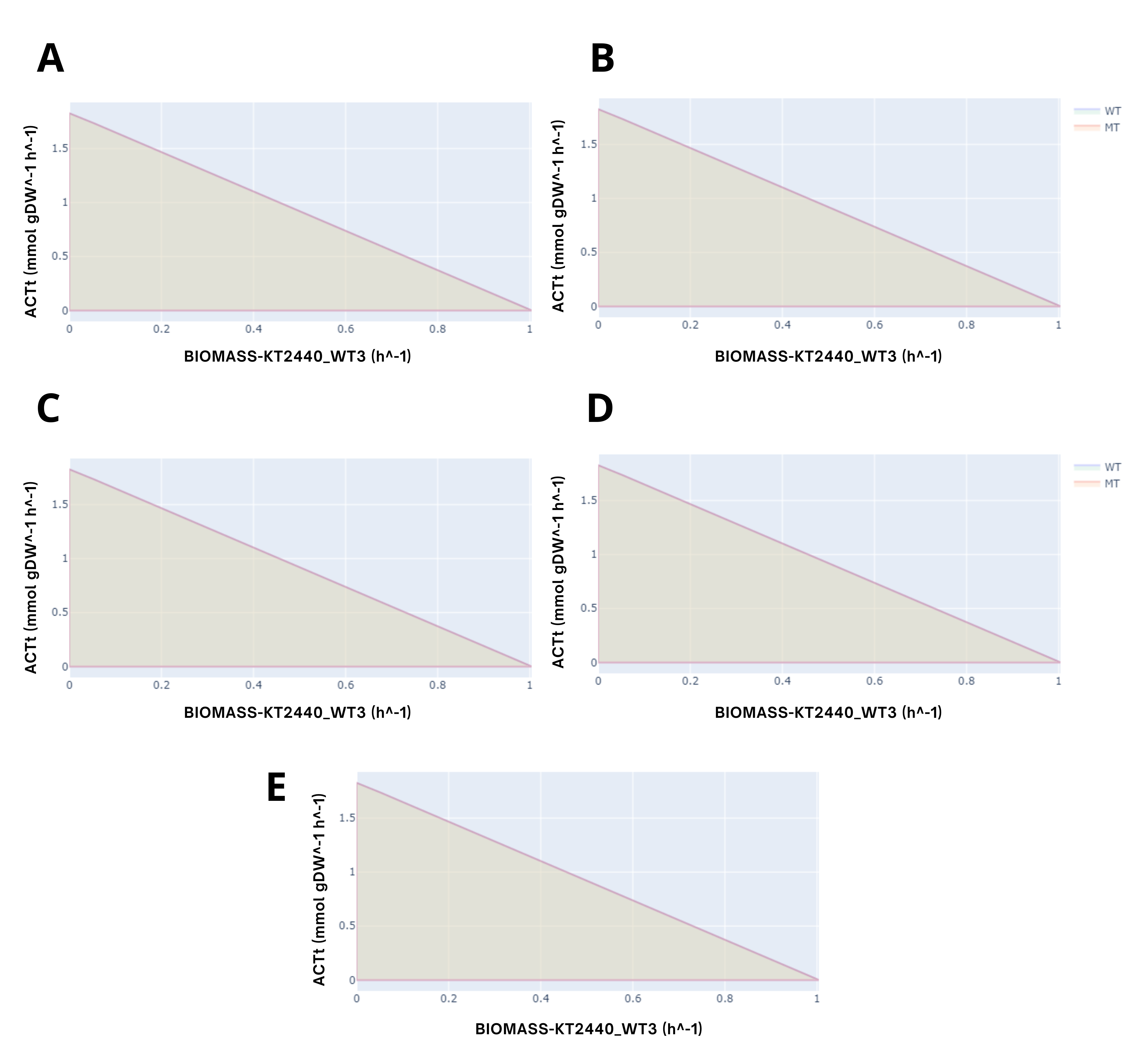


**Figure 4.** Production envelopes of strain designs suggested by OptKnock. Engineering objective/target: ACTt. Carbon source: GLC+MR. (A) FRDO6r knockout (B) RECOAH13 knockout (C) MGCH knockout (D) IMPD knockout (E) PRPPS knockout. WT: iJN1462c; MT: mutant-type/knocked out strain design.

**Production Envelopes of cMCS Strain Designs**


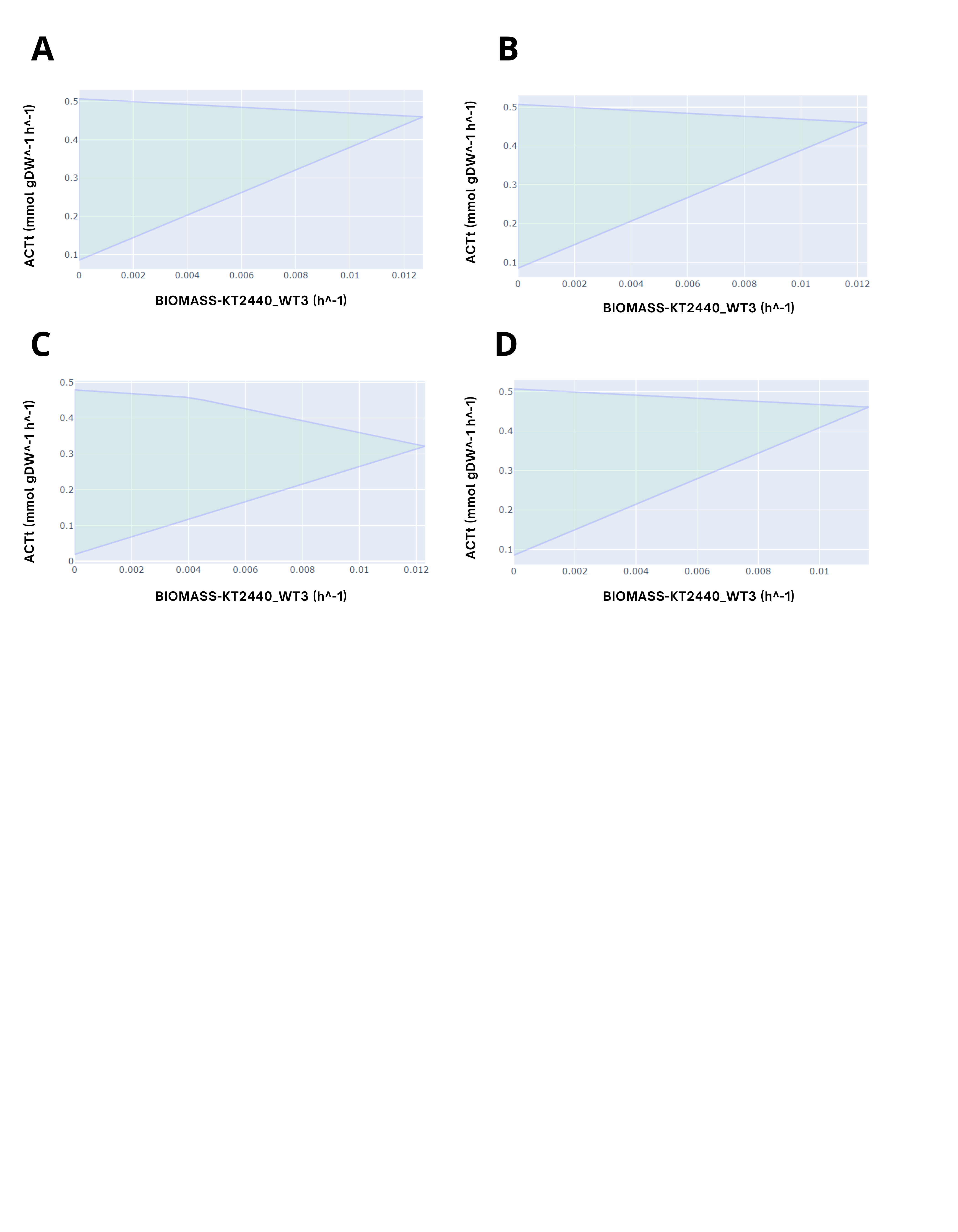


**Figure 5.** Production envelopes of strain designs suggested by cMCS. (A) Strain Design 1, MR. Knockouts: MALS, FDH, SUCD4, ALDD2y, ALDD2x, SUCDi, GLYOX, NACODA, PGL. (B) Strain Design 2, MR. Knockouts: MALS, ALDD2y, MTHFC, ALDD2x, SUCDi, GLYOX, NACODA, ACACt2pp, RPE. (C) Strain Design 4, MR. Knockouts: SUCOAS, HIBDkt, PUTA3, ALDD2y, GLYOX, ACS2, PPS, ACONTb, GLYO1, PGL, ACACt2pp, MDH, ALDD2x, NTD11, FTHFD, PC, GARFT, ICL, IMPD, P5CD. (D) Strain Design 5, MR. Knockouts: ALDD2y, FTHFD, ALDD2x, MDH, MICITDr, GLYOX, ACACT11, MALS, PGL, ACONTa, NACODA.
