# Supplementary material for: Computational analysis into the potential of azo dyes as a feedstock for actinorhodin biosynthesis in Pseudomonas putida
Source: PLoS One. 2024 Mar 4;19(3):e0299128. doi: 10.1371/journal.pone.0299128 (PMC10911627; doi:10.1371/journal.pone.0299128)
Supplement: S5 File — Contains all the native and non-native reactions added to iJN1462c to enable conversion of methyl red to actinorhodin. (DOCX) [file pone.0299128.s005.docx]

**Supporting Information**

**Computational** **Analysis into the Potential of Azo Dyes as a Feedstock for Actinorhodin Biosynthesis in *Pseudomonas putida***

Parsa Nayyara^1, 2, *^, Dani Permana^3, *^, Riksfardini A. Ermawar^4^, Ratih Fahayana^1^

^1^Sekolah Menengah Atas Negeri (SMAN) 5 Surabaya, Jalan Kusuma Bangsa No. 21, Surabaya 60272, Indonesia

^2^University of British Columbia, Vancouver, British Columbia V6T 1Z4, Canada

^3^Research Center for Applied Microbiology, The National Research and Innovation Agency of the Republic of Indonesia (Badan Riset dan Inovasi Nasional (BRIN)), Kawasan Sains dan Teknologi (KST) Ir. Soekarno, Jalan Raya Jakarta-Bogor, KM. 46, Cibinong, Bogor 16911, Indonesia

^4^Research Center for Biomass and Bioproducts, The National Research and Innovation Agency of the Republic of Indonesia (BRIN), Kawasan Sains dan Teknologi (KST) Ir. Soekarno, Jalan Raya Jakarta-Bogor, KM. 46, Cibinong, Bogor 16911, Indonesia

Corresponding Authors:

*E-mail : [nayyara@student.ubc.ca](mailto:nayyara@student.ubc.ca); [dani008@brin.go.id](mailto:dani008@brin.go.id)

**Table S1. Reactions Involved in Methyl Red to Actinorhodin Conversion**

| **Reaction** | **Reaction Description** | **GPR Association** | **Organism of Origin** | **Reference** |
| --- | --- | --- | --- | --- |
| AzoR_MR | Methyl Red [c] + 2 H+ [c] + 2.0 NADH [c] --> N,N-dimethyl-4-phenylenediamine [c] + Anthranilate [c] + 2.0 NAD+ [c] | PP_2866 or PP_4538 | *Pseudomonas putida* KT2440 | [1] |
| AnthDO | Anthranilate [c] + 3.0 H+ [c] + NADH [c] + O2 [c] --> Catechol [c] + CO2 [c] + NAD+ [c] + NH4+ [c] | *ant*ABC | *Acinetobacter*  *baylyi* ADP1 | [2] |
| CAT23DOX | Catechol [c] + O2 [c] --> 2-Hydroxymuconate semialdehyde [c] | pWW0_097 | *Pseudomonas putida* KT2440 | [1] |
| HMSH | 2-Hydroxymuconate semialdehyde [c] + H2O [c] --> Formate [c] + 2.0 H+ [c] + 2-Oxopent-4-enoate [c] | pWW0_095 | *Pseudomonas putida* KT2440 | [1] |
| OP4ENH | 2-Oxopent-4-enoate [c] + H2O [c] --> 4-Hydroxy-2-oxopentanoate [c] | pWW0_094 | *Pseudomonas putida* KT2440 | [1] |
| HOPNTAL | 4-Hydroxy-2-oxopentanoate [c] --> Acetaldehyde [c] + Pyruvate [c] | pWW0_092 | *Pseudomonas putida* KT2440 | [1] |
| ACALD | Acetaldehyde [c] + CoA [c] + NAD+ [c] --> Acetyl-CoA [c] + H+ [c] + NADH [c] | pWW0_093 | *Pseudomonas putida* KT2440 | [1] |
| PDH | CoA [c] + NAD+ [c] + Pyruvate [c] --> Acetyl-CoA [c] + CO2 [c] + NADH [c] | (PP_0339 and PP_0338 and PP_4187) or (PP_0339 and PP_0338 and PP_5366) | *Pseudomonas putida* KT2440 | [1] |
| ACCOAC | Acetyl-CoA [c] + ATP [c] + HCO3 [c] --> ADP [c] + H+ [c] + Malonyl-CoA [c] + Phosphate [c] | PP_1607 and PP_0559 and PP_0558 and PP_1996 | *Pseudomonas putida* KT2440 | [1] |
| ACTS1 | ACPact[c] + Malonyl-CoA [c] <=> CoA [c] + malACPact[c] | SCO5089 | *Streptomyces coelicolor* | [3] |
| ACTS2 | Acetyl-CoA [c]+ H+ [c] + malACPact[c] -> actACPact[c] + CO2 [c] + CoA [c] | SCO5087 and SCO5088 | *Streptomyces coelicolor* | [3] |
| ACTS3 | Malonyl-CoA [c] + H+ [c] + actACPact[c] -> pk6ACPact[c] + CoA [c] + CO2 [c] | SCO5087 and SCO5088 | *Streptomyces coelicolor* | [3] |
| ACTS4 | Malonyl-CoA [c] + H+ [c] + pk6ACPact[c] -> pk8ACPact[c] + CoA [c] + CO2 [c] | SCO5087 and SCO5088 | *Streptomyces coelicolor* | [3] |
| ACTS5 | Malonyl-CoA [c] + H+ [c] + pk8ACPact[c] -> pk10ACPact[c] + CoA [c] + CO2 [c] | SCO5087 and SCO5088 | *Streptomyces coelicolor* | [3] |
| ACTS6 | Malonyl-CoA [c] + H+ [c] + pk10ACPact[c] -> pk12ACPact[c] + CoA [c] + CO2 [c] | SCO5087 and SCO5088 | *Streptomyces coelicolor* | [3] |
| ACTS7 | Malonyl-CoA [c] + H+ [c] + pk12ACPact[c] -> pk14ACPact[c] + CoA [c] + CO2 [c] | SCO5087 and SCO5088 | *Streptomyces coelicolor* | [3] |
| ACTS8 | Malonyl-CoA [c] + H+ [c] + pk14ACPact[c] -> pk16ACPact[c] + CoA [c] + CO2 [c] | SCO5087 and SCO5088 | *Streptomyces coelicolor* | [3] |
| ACTS9 | pk16ACPact[c] -> actint1ACPact[c] | SCO5087 and SCO5088 | *Streptomyces coelicolor* | [3] |
| ACTS10 | actint1ACPact[c] + NADPH [c] + H+ [c] -> actint2ACPact[c] + NADP+ [c] | SCO5086 | *Streptomyces coelicolor* | [3] |
| ACTS11 | actint2ACPact[c] -> actint3ACPact[c] + 2 H2O [c] | SCO5090 | *Streptomyces coelicolor* | [3] |
| ACTS12 | actint3ACPact[c] -> actint4ACPact[c] + H2O [c] | SCO5091 | *Streptomyces coelicolor* | [3] |
| ACTS13 | actint4ACPact[c] + NADPH [c] + H+ [c] -> actint5ACPact[c] + NADP+ [c] | SCO5072 | *Streptomyces coelicolor* | [3] |
| ACTS14 | actint5ACPact[c] -> dnpaACPact[c] + H2O [c] | SCO5071 or SCO5072 or SCO5074 | *Streptomyces coelicolor* | [3] |
| ACTS15 | dnpaACPact[c] + NADPH [c] + H+ [c] -> ddhkACPact[c] + NADP+ [c] | SCO5023 or SCO5075 | *Streptomyces coelicolor* | [3] |
| ACTS16 | ddhkACPact[c] + O2 [c] -> dhkACPact[c] + H2O [c] | (SCO5080 and SCO5092) or SCO5081 | *Streptomyces coelicolor* | [3] |
| ACTS17 | dhkACPact[c] + NADH [c] + H+ [c] + O2 [c] -> hdhkACPact[c] + NAD+ [c] + H2O [c] | SCO5080 and SCO5092 | *Streptomyces coelicolor* | [3] |
| ACTS18 | hdhkACPact[c] + H2O [c] -> hdhk[c] + H+ [c] + ACPact[c] |  | *Streptomyces coelicolor* | [3] |
| ACTS19 | 2 hdhk[c] + NAD+ [c] -> ACT[c] + NADH [c] + H+ [c] |  | *Streptomyces coelicolor* | [3] |
| ACTt | ACT[c] + 2 NAD+ [c] -> gACT[e] + 2 NADH [c] | SCO5083 and SCO5084 | *Streptomyces coelicolor* | [3] |

**References:**

[1] Nogales, J.; Mueller, J.; Gudmundsson, S.; Canalejo, F. J.; Duque, E.; Monk, J.; Feist, A. M.; Ramos, J. L.; Niu, W.; Palsson, B. O. High‐quality Genome‐scale Metabolic Modelling of Pseudomonas Putida Highlights Its Broad Metabolic Capabilities. *Environ Microbiol* 2020, 22 (1), 255–269. <https://doi.org/10.1111/1462-2920.14843>.

[2] Eby, D. M.; Beharry, Z. M.; Coulter, E. D.; Kurtz, J.; Neidle, E. L. Characterization and Evolution of Anthranilate 1,2-Dioxygenase from Acinetobacter Sp. Strain ADP1. *J Bacteriol* 2001, 183 (1), 109–118. <https://doi.org/10.1128/JB.183-1.109-118.2001>.

[3] Amara, A.; Takano, E.; Breitling, R. Development and Validation of an Updated Computational Model of Streptomyces Coelicolor Primary and Secondary Metabolism. *BMC Genomics* 2018, 19 (1). <https://doi.org/10.1186/s12864-018-4905-5>.
