# Supplementary material for: Computational analysis into the potential of azo dyes as a feedstock for actinorhodin biosynthesis in Pseudomonas putida
Source: PLoS One. 2024 Mar 4;19(3):e0299128. doi: 10.1371/journal.pone.0299128 (PMC10911627; doi:10.1371/journal.pone.0299128)
Supplement: S6 File — Evaluation of the strain designs found by the cMCS computation on MR in other common aromatic carbon sources. (DOCX) [file pone.0299128.s006.docx]

**Supporting Information**

**Computational** **Analysis into the Potential of Azo Dyes as a Feedstock for Actinorhodin Biosynthesis in *Pseudomonas putida***

Parsa Nayyara^1, 2, *^, Dani Permana^3, *^, Riksfardini A. Ermawar^4^, Ratih Fahayana^1^

^1^Sekolah Menengah Atas Negeri (SMAN) 5 Surabaya, Jalan Kusuma Bangsa No. 21, Surabaya 60272, Indonesia

^2^University of British Columbia, Vancouver, British Columbia V6T 1Z4, Canada

^3^Research Center for Genetic Engineering, The National Research and Innovation Agency of the Republic of Indonesia (Badan Riset dan Inovasi Nasional (BRIN)), Kawasan Sains dan Teknologi (KST) Ir. Soekarno, Jalan Raya Jakarta-Bogor, KM. 46, Cibinong, Bogor 16911, Indonesia

^4^Research Center for Biomass and Bioproducts, The National Research and Innovation Agency of the Republic of Indonesia (BRIN), Kawasan Sains dan Teknologi (KST) Ir. Soekarno, Jalan Raya Jakarta-Bogor, KM. 46, Cibinong, Bogor 16911, Indonesia

Corresponding Authors:

*E-mail : [nayyara@student.ubc.ca](mailto:nayyara@student.ubc.ca); [dani008@brin.go.id](mailto:dani008@brin.go.id)

**Table of Contents**

**Tables**

Table 1. Flux Distributions on Benzoate

Table 2. Flux Distributions on 4-hydroxybenzoate

Table 3. Flux Distributions on Vanillin

Table 4. Flux Distributions on Vanillate

Table 5. Flux Distributions on Ferulate

Table 6. Flux Distributions on Gallate

**Table 1.** Flux Distributions on Benzoate

| **Strain Design** | **Biomass**  **(mmol/gDW/h)** | **ACT production (mmol/gDW/h)** | **Min. Guaranteed Yield** | **ACT Yield at Optimum Growth** | **BPCY** |
| --- | --- | --- | --- | --- | --- |
| WT | 0.6748 | 0.000 | 0.000 | 0.000 | 0.000 |
| 1 | 0.3575 | 0.000 | 0.000 | 0.000 | 0.000 |
| 2 | 0.3468 | 0.000 | 0.000 | 0.000 | 0.000 |
| 4 | 0.3335 | 0.000 | 0.000 | 0.000 | 0.000 |
| 5 | 0.2980 | 0.000 | 0.000 | 0.000 | 0.000 |

**Table 2.** Flux Distributions on 4-hydroxybenzoate

| **Strain Design** | **Biomass**  **(mmol/gDW/h)** | **ACT production (mmol/gDW/h)** | **Min. Guaranteed Yield** | **ACT Yield at Optimum Growth** | **BPCY** |
| --- | --- | --- | --- | --- | --- |
| WT | 0.6234 | 0.000 | 0.000 | 0.000 | 0.000 |
| 1 | 0.000 | 0.000 | 0.000 | 0.000 | 0.000 |
| 2 | 0.000 | 0.000 | 0.000 | 0.000 | 0.000 |
| 4 | 0.2543 | 0.02752 | 0.000 | 0.003423 | 0.0008707 |
| 5 | 0.1573 | 0.000 | 0.000 | 0.000 | 0.000 |

**Table 3.** Flux Distributions on Vanillin

| **Strain Design** | **Biomass**  **(mmol/gDW/h)** | **ACT production (mmol/gDW/h)** | **Min. Guaranteed Yield** | **ACT Yield at Optimum Growth** | **BPCY** |
| --- | --- | --- | --- | --- | --- |
| WT | 0.8230 | 0.000 | 0.000 | 0.000 | 0.000 |
| 1 | 0.8228 | 0.000 | 0.000 | 0.000 | 0.000 |
| 2 | 0.8068 | 0.000 | 0.000 | 0.000 | 0.000 |
| 4 | 0.5375 | 0.000 | 0.000 | 0.000 | 0.000 |
| 5 | 0.8188 | 0.000 | 0.000 | 0.000 | 0.000 |

**Table 4.** Flux Distributions on Vanillate

| **Strain Design** | **Biomass**  **(mmol/gDW/h)** | **ACT production (mmol/gDW/h)** | **Min. Guaranteed Yield** | **ACT Yield at Optimum Growth** | **BPCY** |
| --- | --- | --- | --- | --- | --- |
| WT | 0.6885 | 0.000 | 0.000 | 0.000 | 0.000 |
| 1 | 0.000 | 0.000 | 0.000 | 0.000 | 0.000 |
| 2 | 0.2068 | 0.000 | 0.000 | 0.000 | 0.000 |
| 4 | 0.4040 | 0.000 | 0.000 | 0.000 | 0.000 |
| 5 | 0.3612 | 0.000 | 0.000 | 0.000 | 0.000 |

**Table 5.** Flux Distributions on Ferulate

| **Strain Design** | **Biomass**  **(mmol/gDW/h)** | **ACT production (mmol/gDW/h)** | **Min. Guaranteed Yield** | **ACT Yield at Optimum Growth** | **BPCY** |
| --- | --- | --- | --- | --- | --- |
| WT | 0.9395 | 0.000 | 0.000 | 0.000 | 0.000 |
| 1 | 0.000 | 0.000 | 0.000 | 0.000 | 0.000 |
| 2 | 0.1155 | 0.000 | 0.000 | 0.000 | 0.000 |
| 4 | 0.3552 | 0.000 | 0.000 | 0.000 | 0.000 |
| 5 | 0.2971 | 0.000 | 0.000 | 0.000 | 0.000 |

**Table 6.** Flux Distributions on Gallate

| **Strain Design** | **Biomass**  **(mmol/gDW/h)** | **ACT production (mmol/gDW/h)** | **Min. Guaranteed Yield** | **ACT Yield at Optimum Growth** | **BPCY** |
| --- | --- | --- | --- | --- | --- |
| WT | 0.9395 | 0.000 | 0.000 | 0.000 | 0.000 |
| 1 | 0.5600 | 0.000 | 0.000 | 0.000 | 0.000 |
| 2 | 0.5043 | 0.000 | 0.000 | 0.000 | 0.000 |
| 4 | 0.5168 | 0.000 | 0.000 | 0.000 | 0.000 |
| 5 | 0.4109 | 0.000 | 0.000 | 0.000 | 0.000 |
