# Supplementary material for: Computational analysis into the potential of azo dyes as a feedstock for actinorhodin biosynthesis in Pseudomonas putida
Source: PLoS One. 2024 Mar 4;19(3):e0299128. doi: 10.1371/journal.pone.0299128 (PMC10911627; doi:10.1371/journal.pone.0299128)
Supplement: Attachment — Submitted filename: PLOS One_Comments.docx [file pone.0299128.s007.docx]

The authors explore the potential of using azo dye methyl red as the sole carbon and energy source for growth and couple it to production of a polyketide in the gram negative soil bacterium *P.putida* and enumerate strain designs for the same using constrained minimal cut sets. The manuscript is well-written, and the idea is novel. While this is theoretically feasible and the simulations quite exhaustive, the lack of a preliminary experimental confirmation to ascertain the potential of the host to use the said azo dye in a cofactor intensive catabolic pathway as a sole carbon source needs to be verified and/or such experimental growth data cross-verified with the simulations (in the wild-type model) before ambitiously pairing it to a polyketide production. The results section was difficult to follow as well due to a lack of comparative/superimposed flux maps for the strain designs. The manuscript can be restructured to make it easily decipherable for the readers. Considering the heavy engineering that needs to go into such a growth coupled strain (>17 gene knockouts or knockdowns) on top of the metabolic burden enforced by the substrate (toxic above a certain threshold) and multistep pathway-based target metabolite production (either on a replicative plasmid or genomically integrated), it might be prudent to demonstrate growth coupling strategies for other industrially important metabolites with simpler pathways. Overall, I would find this manuscript more compelling with some experimental validations.

**Specific comments**

**Introduction**

1. Are there any prior publications with methyl red as the sole carbon and energy source in *P.putida*? What is the threshold limit for this azo dye in *P.putida* or its physiological response characterization? Please cite if any.
2. Why PKSs? Couldn’t you start with simpler metabolites of interest derived from acetyl-coA perhaps for demonstration?
3. Cite some recent PKS studies in *P.putida*.
4. The cMCS designs are specific to the carbon source chosen - need more supportive evidence beyond a single study.

**Materials and Methods**

1. Table 1: Please give the appropriate equations for the reactions.
2. Please add the details of the ACT reactions added in the supplementary with cofactors.
3. Did you use different molar ratios of Glc and MR?
4. Do you have a ATP maintenance on MR?
5. Please mention in detail in supplementary all the reactions added because of the gap-filling process.
6. Could you also give the relevant cofactor fluxes (production/consumption) for the 4 strain designs?
7. cMCS computation Preprocessing: Mention the details of A-E in the supplementary for the preprocessing of the model so that the readers can reproduce it if need be. It is good to mention in the main text the stats for the same (x total reactions, y removed from each category or in %). Are the “lethal phenotypes” pertaining to biomass formation or conditionally essential reactions with respect to substrates such as aromatics in this case (from Rb-TnSeq fitness data etc)?
8. cMCS computations: What does 0% biomass and 0% ACT mean? What are you simulating there? Shouldn’t you be starting with 0 and 1% respectively for growth coupling?
9. Expand BPCY at the first mention and cite Patil et al
10. Cut-off of 25 gene knockouts still seems to be high. Do you have experimental evidences of such a high number of gene knockouts or knockdowns in *P.putida*?
11. What is a “minimum guaranteed ACT yield”? 1% of Tmax

**Results and Discussion**

**3.1**

1. There are other routes for anthranilate degradation (to Trp for instance). Not including ACT, does MR support growth in the first place? Does it still need AnthDO for that?

2. “Native and non-native reactions from..” – Please list the reactions in supplementary section

3. Fig 1 is not high resolution. Hard to read. Could you perhaps also provide a simple pathway figure (not with Escher) with cofactors etc? Is the glyoxylate cycle not operational.

4. Although the simulations show the catabolism through the meta cleavage pathway (atleast in Fig 1), experimental observations of aromatics degradation in *P.putida* have shown that both or either of the two pathways could be active (such as ortho- and meta- for benzoate). How is ortho over meta favorable?

Table 2: Please show the gene knockouts on a metabolic map.

**3.3**

1. “Previously, we have attempted to use both Optknock and OptGene to..”, is there a prior publication from the group based on this?

**3.4**

**1.** “It is worth noting that growth-coupled designs for ACT were only found when MR is present as a carbon source, and none was found in the GLC-only medium” – why do you think there were no growth couple regimes on glucose?

2. Please rephrase the entire section. It is too wordy and difficult to go back and forth.

Fig 4 and 5: Why were the growth coupled regimes optimized for growth rather than using 1% of both biomass and product ?

How is acetaldehyde produced in strain 4? From acetyl CoA? Isn’t that futile/cyclic?

PP_0321 : L-Threonine aldolase reaction is thermodynamically favored in the forward reaction (Biocyc). Would it still work in the reverse for this strain?

L-threonine → acetaldehyde + glycine

“Although growth-coupling regimes exist for three of the tested aromatic compounds,…”, I do not see any values for ACT on the aromatics except for one. Could you please explain in detail?

**3.5**

Does the growth coupling still hold when you optimize for growth in the strain designs?

Fig 7a: Does the first blue bar for WT represent the theoretical max Act yield?

**Conclusion**

Considering you are advocating for MR from wastewater or dye industry effluents, how feasible, cost effective and sustainable do you think it is to supply this as a feedstock to a large-scale microbial fermentation process? Does the MR have to be separated first as other substrates in the wastewater might uncouple production.
