# Supplementary material for: Computational analysis into the potential of azo dyes as a feedstock for actinorhodin biosynthesis in Pseudomonas putida
Source: PLoS One. 2024 Mar 4;19(3):e0299128. doi: 10.1371/journal.pone.0299128 (PMC10911627; doi:10.1371/journal.pone.0299128)
Supplement: Attachment — Submitted filename: Response Letter_2nd Revision.docx [file pone.0299128.s009.docx]

**Overall Response to the Reviewers:**

We express our appreciation for the valuable comments raised by the reviewers. We have carefully revised the manuscript in accordance with their suggestions. We have attached a detailed point-by-point response to the comments raised by each reviewer. These responses describe the changes we made to the manuscript. We believe that the revised manuscript has been improved satisfactorily and hope that this version is now acceptable for publication in *PLOS ONE*.

**Point-by-point Responses to the Comments Raised by the Reviewers**

**Reviewer: 1**

**Recommendation:** Publish after major revisions.

**General Comment:**

*The manuscript by Nayyara and coworkers explores the potential of using azo dye methyl red as the sole carbon and energy source for growth and couple it to production of a polyketide in the gram-negative soil bacterium P.putida and enumerate strain designs for the same using constrained minimal cut sets. The manuscript is well-written, and the idea is novel.* *While this is theoretically feasible and the simulations quite exhaustive, the lack of a preliminary experimental confirmation to ascertain the potential of the host to use the said azo dye in a cofactor intensive catabolic pathway as a sole carbon source needs to be verified and/or such experimental growth data cross-verified with the simulations (in the wild-type model) before ambitiously pairing it to a polyketide production. The results section was difficult to follow as well due to a lack of comparative/superimposed flux maps for the strain designs. The manuscript can be restructured to make it easily decipherable for the readers. Considering the heavy engineering that needs to go into such a growth coupled strain (>17 gene knockouts or knockdowns) on top of the metabolic burden enforced by the substrate (toxic above a certain threshold) and multistep pathway-based target metabolite production (either on a replicative plasmid or genomically integrated), it might be prudent to demonstrate growth coupling strategies for other industrially important metabolites with simpler pathways. Overall, I would find this manuscript more compelling with some experimental validations.*

**Response to reviewer #1:**

We thank reviewer #1 for providing thoughtful questions, suggestions, and recommendations that have helped strengthen our arguments and make the manuscript more comprehensible for readers. We have revised our manuscript and restructured the results section to make it easier for readers to follow.

As pointed out by reviewer #1, we recognize the lack of experimental data to confirm the predictions generated in this study and the ambitious engineering that needs to go into creating the strain designs. In our revised manuscript we have included previous studies that have experimentally confirmed the possibility for *P. putida* to degrade methyl red and reported the physiological limits and toxicity of azo dyes against *P. putida*. However, due to limited funding and access to resources that would allow us to further test the production of actinorhodin and build the growth-coupled strain designs predicted, we are unable to provide further experimental validations in this paper. We apologize for not meeting your expectations at this stage.

Despite the heavily computational nature of this study, we still believe that this paper provides important insights into how influential the choice of carbon sources is in altering the possible flux spaces accessible by the system. We hope that this study inspires other researchers that do have the resources to conduct experimental validations based on the designs reported, or at least, consider the conclusions made in this study when choosing a carbon source for bioproduction or valorization of waste into an industrially important metabolite. Our detailed response and revisions made in accordance with each comment raised by reviewer #1 have been provided below.

**1.1 Introduction**

**Specific comment-1.1.1**

*Are there any prior publications with methyl red as the sole carbon and energy source in P.putida? What is the threshold limit for this azo dye in P.putida or its physiological response characterization? Please cite if any.*

**Answer-1.1.1**

We thank reviewer #1 for raising this point, we realized that it was not mentioned specifically in the manuscript that there are previous studies that have indicated *P. putida’*s ability to physiologically withstand and decolourize up to 250 µM of methyl red (Mendes et al. 2011) and other common azo dyes. We have revised the manuscript and added new sentences and referenced those studies as follows.

***Please see Page-5, line-14.***

Biodegradation of simple azo dyes such as methyl red has previously been observed in *P. putida* MET94 and *P. putida* OsEnB_HZB_G20. *P. putida* MET94 was selected from 48 bacterial strains for its ability to decolourize a range of common azo dyes to a higher extent compared to other strains. For methyl red, although specific growth rates decreased as methyl red concentration increased (indicating some level of toxicity), *P. putida* MET94 decolourized 90% of the dye under 24 h when grown in LB-rich media supplemented with 150 µM of the dye. The azoreductase (PpAzoR) found in *P. putida* MET94 had a 99% sequence similarity with an azoreductase found in *P. putida* KT2440.

However, we have not found any evidence elucidating a pathway in *P. putida* KT2440 to utilize azo dye (i.e., methyl red) as the sole carbon source. Based on our gap-filling analysis (Section 3.1) we identified that only 1 reaction is needed to connect a gap between the anthranilate node to the rest of the aromatic degradation pathway, and consequently the central carbon metabolism. We have also elaborated that *P. putida* has a native benABC operon encoding benzoate dioxygenase which is predicted to use not only benzoate but also anthranilate as a substrate to produce catechol, an intermediate in the aromatic catabolic pathway. However, not enough biochemical evidence is available to confidently assume this substrate usage. Thus, we included anthranilate-1,2-dioxygenase as one of the non-native reactions to be heterologously expressed to enable our synthetic pathway in iJN1462c. The addition of this pathway would theoretically enable the catabolism of methyl red and its aromatic amine, anthranilate, simultaneously providing a mechanism for *P. putida* KT2440 to detoxify aromatic amines from azo dye cleavage.

**Specific comment-1.1.2**

*Why PKSs? Couldn’t you start with simpler metabolites of interest derived from acetyl-coA perhaps for demonstration?*

**Answer-1.1.2**

We thank reviewer #1 for their thoughtful questions. We chose PKSs for several reasons. 1) They are economically and medicinally valuable compounds; 2) there have been recent efforts in engineering PKSs as they are modular and highly engineerable to produce specialized chemicals not found in nature; 3) they are an example of a target metabolite that is known to be difficult to growth-couple as they require specific, multi-reaction pathways that uses a considerable share of the carbon flux; and 4) actinorhodin specifically is an example of a polyketide with an oxidation step that produces excess NADH. Excess NADH is speculated to be a reason for growth-coupling infeasibility on glucose because it creates a redox imbalance.

From our preliminary work using OptKnock and OptGene, the results of which we have included in **Supporting Information 4**, 0 mmol/gDW/h of actinorhodin can be produced when growth was the objective and actinorhodin was shown to be difficult and even infeasible to growth-couple. Thus, actinorhodin was chosen to demonstrate that altering the carbon substrate can make a metabolite previously infeasible to growth-couple possible to couple. More specifically in our case, metabolites that cannot be growth coupled anRegd happen to also produce excess NADH can benefit from using substrates like azo dyes that consume NADH.

***Please see Page-22, line-6.***

Previously, we have attempted to use both Optknock and OptGene to generate knockout suggestions that optimize the MR to ACT pathway (Supporting Information 4, Figure 1-4). Solutions suggested by Optknock enabled very weak growth coupling (Supporting Information 4, Figure 1-4) and OptGene found no feasible knockout targets.

We also extended a sentence to clarify the above point:

***Please see Page-6, line-12.***

Based on these studies and with the growing interest to transition from sugar-based substrates, we were interested to see if there are any differences in the cMCS solution space for a heterologous product when using glucose versus a non-sugar-based substrate, especially specialized products like actinorhodin that are difficult to growth-couple. So far, the cMCS approach has not been used to optimize the utilization of non-traditional aromatic carbon sources like azo dyes to produce a heterologous product.

Although starting with acetyl-CoA, a precursor to actinorhodin, would be a reasonable test to see if conversion from azo dyes into actinorhodin is feasible, a certain level of acetyl-CoA was already produced even when growth was set as the objective (data not shown in the manuscript), meaning that acetyl-CoA is required for growth and is already growth-coupled (albeit not a strong growth-coupling type). Our aim was simply to see if a naturally uncoupled metabolite can be coupled by changing the carbon source—the question of how to turn a weak coupling phenotype into a strong coupling phenotype requires a separate study. Moreover, acetyl-CoA is a central metabolite connected to many branch points in the cell’s metabolism, so there are more cofactors to account for than NADH when redirecting fluxes to acetyl-CoA. A carbon source with different properties than azo dyes is likely needed to strongly growth-couple acetyl-CoA.

**Specific comment-1.1.3**

*Cite some recent PKS studies in P.putida.*

**Answer-1.1.3**

We thank reviewer #1 for this suggestion. We have added new sentences and cited several studies as references to demonstrate the possibility of expressing and engineering PKSs in *P. putida* KT2440.

***Please see Page-5, line-9.***

Recent studies have demonstrated heterologous expression of PKSs in *P. putida* KT2440 with varying performances, either on par or better than the native host and some inferior to other production hosts.

**Specific comment-1.1.4**

*The cMCS designs are specific to the carbon source chosen - need more supportive evidence beyond a single study.*

**Answer-1.1.4**

We thank reviewer #1 for the suggestion. In the introduction, we mentioned a study from Banerjee et al. (2020) which shows indications that “cMCS suggested designs are specific to the carbon source chosen”, because their suggested knockout sets for growth-coupling of indigoidine production from glucose did not produce the same growth-coupled phenotype prediction when the carbon source was changed to *p*-coumarate and lysine. Up until this point, Banerjee et al’s study is the only one we found that has tested and observed that the same cMCS knockout set for the same target product produced different results in different carbon sources. We have added one other reference, (Kamp & Klamt, 2017) speculating that infeasible growth-coupling may become feasible when substrate is changed, in our introduction as follows.

***Please see Page-6, line-8.***

Another study by Kamp and Klamt (2017) has found that growth-coupling was possible for almost all native metabolites in five production hosts with glucose as substrate, and expected that for some metabolites unfeasible to couple, changing carbon substrate may make growth-coupling feasible; however, this prediction was not tested further.

We completely agree with reviewer #1 that more evidence is needed to confirm this, which is one of the reasons why this study was conducted. We saw this interesting cMCS result on *p-*coumarate and lysine in Banerjee et al.’s study that was not elaborated further in their paper. Hence in our study, we attempted to test if changing the carbon substrate (glucose, methyl red, and 6 other aromatic carbon sources) changes the solution space for a heterologous product, and if this can be utilized as a strategy to growth-couple products that are previously predicted to be difficult to growth-couple.

**1.2 Materials & Methods**

**Specific comment-1.2.1**

*Table 1: Please give the appropriate equations for the reactions.*

**Answer-1.2.1**

We thank reviewer #1 for this valuable suggestion. We did not include the equations in Table 1 since reaction formulas are retrievable from the model (Supporting Information 1) in COBRA/COBRApy. However, we have decided to revise the manuscript and include the reaction description and equation in Table 1 for clarity. We believe this also answers comment 2.5 by reviewer #2.

**Specific comment-1.2.2**

*Please add the details of the ACT reactions added in the supplementary with cofactors.*

**Answer-1.2.2**

We thank reviewer #1 for the valuable comments and suggestions. We have revised the Supporting Information sections and added all reactions (native and non-native) involved in the methyl red to actinorhodin synthetic pathway in Supporting Information 5, with details about the reaction formula, stoichiometry, and organism of origin. We believe this also answers comment-1.3.2.

**Specific comment-1.2.3**

*Did you use different molar ratios of Glc and MR?*

**Answer-1.2.3**

We thank the reviewer #1 for their question. Genome-scale modelling and flux balance analysis is a method based on stoichiometric ratios and flux distributions at a steady state, no reaction kinetics are involved. Therefore, it is a static, not dynamic, representation of flux distributions. We can only gain insights and retrieve information about the fluxes or intracellular reaction rates (measured in mmol/gDW/hour) at a snapshot of time, but we cannot conclude or input any information about the molar concentrations. Thus, when determining the *in silico* medium, uptake rates are typically used in genome-scale modelling studies instead of molar concentrations of each substrate. Supplying the same molar concentrations of carbon sources does not always mean that the cell is consuming both substrates at the same uptake rates. Uptake rates are a measure of how much substrate the cell can physiologically consume at a steady state, which is influenced by the cell’s stoichiometric flux distributions and regulatory systems. Genome-scale modelling can give information on the former but not the latter, which is a known limitation of this approach. Therefore, in ideal circumstances, one would measure uptake rates experimentally to account for this limitation. We recognize the importance of determining uptake rates as this determines the results of the analyses.

Glucose uptake rate was determined following Nogales et al.’s in silico M9 minimal media + glucose, where it has been measured experimentally. MR uptake rate was kept unbound as there are no previous studies to date that have measured MR uptake rate experimentally in *P. putida* KT2440. However, the rest of the model is well-constrained enough (meaning that all other uptake rates and stoichiometric constraints are clearly defined) that the minimum and maximum MR uptake rate possible stoichiometrically is not infinite but will be limited by the stoichiometric capabilities of the cell. This means that rather than treating MR uptake rate/consumption as a constant (which will require experimental measurement), we treated it as a variable, just like biomass/ACT production. This approach allowed us to observe how much MR the cell can theoretically uptake with changing objectives and media compositions, which we tested through flux balance analysis and flux variability analysis. This is elaborated in detail in Section 2.4 as follows.

***Please see Page-9, line-4.***

Uptake rates for all nutrients were set according to the in silico M9 minimal medium as specified by Nogales et al., except for methyl red (MR), because its specific steady-state uptake rate in P. putida had not been tested experimentally before. Thus, because we aim to analyze the maximum theoretical capability of iJN1462c to consume MR in this study, the MR uptake rate in the medium, if any, was set to 1000 mmol gDW-1 h-1. The value 1000 was chosen to simulate an extreme case in which MR uptake was not constrained and the cell was free to consume as much MR as possible under the constraints of the model.

**Specific comment-1.2.4**

*Do you have an ATP maintenance on MR?*

**Answer-1.2.4**

We thank reviewer #1 for raising this comment. We are not completely certain if this question is asking about the flux-sum of ATP on MR media or about whether consuming MR requires ATP. We have calculated and displayed the cofactor flux-sum for ATP when grown on MR only, GLC only, and MR + GLC under biomass and ACT production as objective; this data is shown in Figure 3. Additionally, consuming MR does not require ATP nor ADP, in contrast to glycolysis which produces ATP as elaborated in page-21, line-1. Despite this, we still see some ATP flux-sum because ATP still needs to be generated by other means to support growth. On MR, ATP generation does not happen in MR catabolism, but further down in the TCA cycle. For this reason, we expected a lower ATP turnover and demand needed to support growth on MR than on GLC (see page-21, line-7). We welcome further clarification and questions if needed.

**Specific comment-1.2.5**

*Please mention in detail in supplementary all the reactions added because of the gap-filling process.*

**Answer-1.2.5**

We thank reviewer #1 for the valuable suggestions. The only reaction added during manual gap-filling was AnthDO and this reaction has been included in Supplementary Information 5.

***Please see Page-16, line-13.***

To complete this reaction gap in the synthetic pathway, a non-native anthranilate-1,2-dioxygenase reaction (AnthDO) from *Acinetobacter sp*. encoded by the *ant*ABC operon was added.

**Specific comment-1.2.6**

*Could you also give the relevant cofactor fluxes (production/consumption) for the 4 strain designs?*

**Answer-1.2.6**

We thank reviewer #1 for providing valuable suggestions. We conducted the cofactor flux-sum analysis to investigate why MR consumption was competing with growth above the observed thresholds and why the MR consumption threshold was lower in GLC+MR than in MR only. The flux-sum analyses revealed that the competition was due to MR requiring NADH to be consumed, however the analysis also found that using GLC as carbon source requires more ATP to be available in the system than using MR as carbon source. This explains our observations in Figure 2. Flux-sums provide an idea of how much a certain metabolite is circulating in the system, and this depends mostly on the carbon source used and the objective of the cell.

Although there might be minute differences in the cofactor flux-sums of iJN1462c and the 4 strain designs, we believe that flux-sums will not provide any meaningful conclusions for the comparison between the 4 strain designs since all four were generated by MCS under the same carbon source (i.e., MR) and all four are evaluated under the same objective (i.e., biomass). Moreover, the quality of a strain design is typically evaluated based on the product yield it can generate and BPCY. Providing flux-sum data would only give insight into how much ATP is demanded by each strain design (which should be similar given the same carbon source and objective), but it would not explain how the strain design is able to support both growth and ACT production—this is evaluated using the pathway utilization analysis. For this reason, please accept our decision to not include flux-sum data for the strain designs in the final manuscript to eliminate redundancy.

**Specific comment-1.2.7**

*cMCS computation Preprocessing: Mention the details of A-E in the supplementary for the preprocessing of the model so that the readers can reproduce it if need be. It is good to mention in the main text the stats for the same (x total reactions, y removed from each category or in %). Are the “lethal phenotypes” pertaining to biomass formation or conditionally essential reactions with respect to substrates such as aromatics in this case (from Rb-TnSeq fitness data etc)?*

**Answer-1.2.7**

We appreciate the suggestions and recommendations provided by reviewer #1. We believe that steps A-E have included all the details needed for readers to determine what reactions to remove from the model using COBRA. There are specialized functions in COBRApy to eliminate blocked reactions and essential reactions. As explained, blocked reactions mean all reactions that were unable to carry flux in any scenario and essential reactions are determined based on FBA on each respective mediums (if the deletion caused biomass flux to be zero, it is an essential reaction). Non-gene-associated reactions are all reactions that have not been assigned a gene association in the model. Transport and peripheral reactions are all reactions in the transport subsystem. Boundary reactions are also removed as they are not biologically meaningful and are just added to allow the substrate to “move” in and out of the model. Please accept our decision not to add any more details to this section to avoid redundancy.

**Specific comment-1.2.8**

*cMCS computations: What does 0% biomass and 0% ACT mean? What are you simulating there? Shouldn’t you be starting with 0 and 1% respectively for growth coupling?*

**Answer-1.2.8**

We thank reviewer #1 for the valuable questions. When performing cMCS calculations, we systematically start with the most relaxed scenario possible, the scenario that enforces the least constraints on the cell, which is 0% biomass and 0% ACT. This is because we do not know if a growth-coupling regime exists, and if it is not feasible in the most relaxed scenario, it will not be feasible in scenarios with added constraints. In cMCS computation, we set two thresholds that define the target region (the region that must be made infeasible by the MCS) and the desired region (the region that needs to be protected by the MCS, i.e., the MCS must enable this region or more). In our case, the target region was any ACT under our minimum threshold; the desired region was any biomass under the threshold.

0% ACT/biomass means that we do not enforce any regions in the flux space to be suppressed or protected, which means the system is free to produce any ACT/biomass or none. In our study, these scenarios act as a control and sanity check that there should always be an MCS that exist under these constraints if growth-coupling is feasible. If, for example, we had started with 0% biomass and 1% ACT, and the computation returns no feasible growth-coupling regimes, it does not necessarily mean that there are no growth-coupling regimes at 0% biomass and 0-1% ACT. Having the data that shows that there is at least 1 MCS at 0% biomass and 0% ACT shows that the system can produce a growth-coupling regime that guarantees somewhere between 0-1% ACT. An example of this would be the calculations on MR + GLC, where 0% and 0% return a solution but 0% and 1% (and vice versa) do not. By contrast, on GLC only, cMCS does not return any regimes at 0% and 0% indicating that we can be fairly certain that growth-coupling is infeasible, even at very low ACT thresholds.

**Specific comment-1.2.9**

*Expand BPCY at the first mention and cite Patil et al*

**Answer-1.2.9**

We thank reviewer #1 for this suggestion. We have revised the manuscript accordingly.

**Specific comment-1.2.10**

*Cut-off of 25 gene knockouts still seems to be high. Do you have experimental evidences of such a high number of gene knockouts or knockdowns in P.putida?*

**Answer-1.2.10**

We thank reviewer #1 for the thoughtful question. We chose 25 as an arbitrary maximum cut-off as there have been examples of knockouts of up to 19 genes done in [other organisms](https://www.ncbi.nlm.nih.gov/pmc/articles/PMC4495200/) although it has not been demonstrated in *P. putida.* Despite this, we have seen studies successfully using multiplex CRISPRi knocking down up to 9 genes (e.g., Banerjee et al.) and knockouts up to 16 genes with RecET-based recombineering (Choi et al) in *P. putida* KT2440. The strain designs that perform best in our analysis (strain designs 2 and 5, see Section 3.5) all have a knockout size of under 19.

**Specific comment-1.2.11**

*What is a “minimum guaranteed ACT yield”? 1% of Tmax*

**Answer-1.2.11**

We thank reviewer #1 for raising this question. The minimum guaranteed ACT yield is the minimum ACT yield that the cell must produce as a result of the knockouts suggested by cMCS. It is not defined as 1% of the theoretical maximum yield, but rather the minimum ACT yield calculated by FVA after the knockout suggestions have been simulated in the strain design.

**1.3 Results & Discussion**

**Specific comment-1.3.1**

*There are other routes for anthranilate degradation (to Trp for instance). Not including ACT, does MR support growth in the first place? Does it still need AnthDO for that?*

**Answer-1.3.1**

We thank reviewer #1 for raising thoughtful comments and questions. In Section 3.1, we have elaborated that without anthranilate-1,2-dioxygenase (AnthDO), the model was unable to grow on MR alone, indicating that there is a gap to be filled in the MR degradation pathway. We recognized the presence of the anthranilate degradation pathway to tryptophan and have investigated why it may not support growth (data not included in the paper). Briefly, although the model can use tryptophan as a sole carbon source, the conversion of anthranilate to tryptophan requires a supply of serine. To use anthranilate, and hence MR, as sole carbon source without AnthDO, the cell needs to either synthesize serine from another carbon source or have an external supply of L-serine from the media. We have tried simulating this in the model—supplying anthranilate only, anthranilate + serine, and anthranilate + glucose to the model without AnthDO. The cell can only grow with anthranilate + serine or anthranilate + glucose. Therefore, although *P. putida* can theoretically degrade anthranilate, the cell still could not use it as a sole carbon source without AnthDO.

***Please see Page-14, line-10.***

This modified version of the model, denoted iJN1462b, was still unable to grow and produce ACT from MR alone due to missing reactions between the anthranilate node and the central carbon metabolism.

**Specific comment-1.3.2**

*“Native and non-native reactions from..” – Please list the reactions in supplementary section*

**Answer-1.3.2**

We thank the reviewer for the valuable recommendations. We have revised the Supporting Information and included these reactions in Supporting Information 5.

**Specific comment-1.3.3**

*Fig 1 is not high resolution. Hard to read. Could you perhaps also provide a simple pathway figure (not with Escher) with cofactors etc? Is the glyoxylate cycle not operational.*

**Answer-1.3.3**

We thank reviewer #1 for raising this concern. We have remade Figure 1 with simpler graphics (without escher) and higher resolution showing the azo dye to actinorhodin pathway in context other central metabolic pathways in *P. putida* (Revised Figure 1 shown below).

Not all cofactors are displayed, for clarity and to avoid overcrowding the figure, however, relevant cofactors in the MR-to-ACT pathway are shown. The highlighted pathway is meant to show the reactions involved in the MR-to-ACT pathway; highlighted reactions do not show if they are operational as this differs between strain designs. For more information on whether a pathway is operational / active on a strain design, please refer to the pathway utilization analysis (Figure 5 and 6).

**Specific comment-1.3.4**

*Although the simulations show the catabolism through the meta cleavage pathway (at least in Fig 1), experimental observations of aromatics degradation in P.putida have shown that both or either of the two pathways could be active (such as ortho- and meta- for benzoate). How is ortho over meta favorable?*

**Answer-1.3.4**

We appreciate the questions raised by the reviewer. FBA simulations indicated utilization of both catechol degradation pathways; meta-cleavage in strain designs 1, 2, and 5, and ortho-cleavage in strain design 4. Without gene knockouts, iJN1462c seems to favor the meta- over ortho- pathway, as shown in Figure 1. In iJN1462, the meta-cleavage pathway requires only 5 reaction steps from catechol to acetyl-CoA, while the ortho-cleavage pathway requires 7 reactions. Therefore, based on FBA and genome-scale modelling, meta- will be favored since it is the shortest path to the central metabolism. In the actual cell, certain regulatory mechanisms may be at play that determines whether the meta- or ortho-cleavage pathway will be favored as explained in experimental studies. However, genome-scale modelling is based only on stoichiometry and does not take into account any thermodynamics, kinetics, or regulatory pathway, which is a known limitation of the approach. Hence, certain pathway preference is typically due to a shorter pathway length, or which one maintains the stoichiometric balance of the system. Certainly, this does not eliminate the possibility that ortho- may be preferred over meta- in the actual cell due to induction by some regulatory factors.

**Specific comment-1.3.5**

*Table 2: Please show the gene knockouts on a metabolic map.*

**Answer-1.3.5**

We thank the reviewer for the valuable suggestion. We have added Figure 4 to show the recommended gene knockouts from each strain design. Gene knockouts are displayed on the same metabolic map as Figure 1 for easier comparison with the unmodified iJN1463c. The new figure is shown below (page-26 in the manuscript).

**Specific comment-1.3.6**

*“Previously, we have attempted to use both Optknock and OptGene to..”, is there a prior publication from the group based on this?*

**Answer-1.3.6**

We thank the reviewer #1 for the thoughtful question. There is no prior publication on this matter. This particular sentence was referring to a preliminary attempt at growth-coupling that we have done prior to using cMCS. Although OptKnock and OptGene failed to suggest any knockout targets for growth-coupling actinorhodin, this first attempt produced valuable data that we built upon in this study with cMCS; we have included this preliminary data in Supporting Information 4. Our reasoning for switching to using cMCS instead of OptKnock and OptGene has also been elaborated on page-23, line-12.

**Specific comment-1.3.7**

*“It is worth noting that growth-coupled designs for ACT were only found when MR is present as a carbon source, and none was found in the GLC-only medium” – why do you think there were no growth couple regimes on glucose?*

**Answer-1.3.7**

We thank the reviewer #1 for the interesting comments and questions. We have discussed why this happens at length in Section 3.4 and have also performed further computational analysis (i.e. the pathway utilization analysis) to confirm our hypotheses. Briefly, glucose catabolism through glycolysis produces a surplus of NADH while methyl red requires NADH to be catabolized. Since actinorhodin biosynthesis also generates excess NADH, using glucose only does not allow the possibility to ‘create’ more NADH demand that forces actinorhodin biosynthesis to be essential for growth. In other words, the cell can and will stop producing actinorhodin and still have ample NADH supply to support growth. Contrarily, with MR, it is possible to make manipulations that increase the already existing NADH demand for MR catabolism. Without glucose to supply NADH, the cell can now be forced to use actinorhodin biosynthesis to support growth on MR only.

***Please see Page-26, line-14.***

We tested this hypothesis in iJN1462c by looking at the fluxes channeled through several key metabolites that represent major pathways in *P. putida*’s carbon metabolism (Figure 5, 6, and 7). Examining which metabolites are produced, thus which pathways are utilized or deactivated when the optimization objective is BIOMASS_KT2440_WT3 versus ACT, reveals where the carbon flux is needed to maximize growth or ACT production. Fluxes in the growth-coupled cMCS designs were also compared to see how fluxes can be distributed, and/or segregated, to support both objectives.

**Specific comment-1.3.8**

*Please rephrase the entire section (3.4). It is too wordy and difficult to go back and forth.*

**Answer-1.3.8**

We appreciate the reviewer for the recommendation and for raising this concern. We have revised the section accordingly without eliminating meaningful information. We hope these changes help improve the clarity and conciseness of the text.

**Specific comment-1.3.9**

*Fig 4 and 5: Why were the growth coupled regimes optimized for growth rather than using 1% of both biomass and product?*

**Answer-1.3.9**

We thank the reviewer #1 for the thoughtful question. In the pathway utilization analysis, our goal is to examine how the flux distribution strategies differ between the WT and the suggested strain designs. Specifically, how the growth-coupled strain designs could still produce ACT when optimizing growth. We calculated WT under both objectives because unlike the growth-coupled designs, WT is not capable of producing both at once; but we wanted to see where in the metabolic network carbon flux was needed when the cell is optimizing growth vs when optimizing ACT.

To find knockout targets that guarantee 1% biomass or product, cMCS enforced the 1% threshold; this is an artificial computational constraint needed to find the solution. Once cMCS identified the knockouts needed to guarantee this threshold, we can implement the knockouts to create a growth-coupled strain design. When these knockouts are implemented in the wet lab, growth would be the main selective pressure because there will be no outside pressure for the cell to maintain 1% ACT. This assumption is commonly used in flux balance analyses since cells that grow faster tend to outcompete other phenotypes, hence cells will always optimize towards growth. This is also the reason why designing a growth-coupled ACT production phenotype is important, because coupling ACT production with growth allows the selection of ACT producing strains by adaptive laboratory evolution (ALE). The aim is to create a strain design with the knockouts that can maintain this guaranteed ACT production even with growth as the objective. The pathway utilization analysis depicted in Fig. 4 and 5 simply tries to understand how exactly the knockouts implemented allowed the cell to rewire its metabolism to support ACT production under biomass as objective, hence, growth was optimized and not 1% act/biomass. In section 3.5, the calculation of product yield and BPCY was also performed under biomass (not ACT) as the objective after the knockouts were simulated for the same reason.

We believe this also answers comment 1.3.12. We have added new sentences to clarify the point above.

***Please see Page-12, line-3.***

The analysis was performed under biomass as the objective to simulate real-life scenario where the cell would be optimizing for growth. For comparison, WT was analyzed under both biomass and ACT production as objective to examine where fluxes need to be channeled to support ACT or biomass production.

***Please also see Page-12, line-19.***

Each reaction knockout set was verified with standard FBA analysis under biomass as the objective and FVA was implemented to ensure guaranteed ACT production even when cells do not grow (i.e., minimum ACT yield is not 0).

**Specific comment-1.3.10**

*How is acetaldehyde produced in strain 4? From acetyl CoA? Isn’t that futile/cyclic?*

*PP_0321: L-Threonine aldolase reaction is thermodynamically favored in the forward reaction (Biocyc). Would it still work in the reverse for this strain? L-threonine → acetaldehyde + glycine*

**Answer-1.3.10**

We appreciate the comments and questions raised by the reviewer #1. In strain design 4, FBA predicted a change in directionality in ACALD, which means acetaldehyde is produced from acetyl-CoA. Futile cycles occur when the reaction is proceeding in both directions simultaneously, this is not the case in strain design 4, as ACALD is predicted to only proceed in the reverse direction. The next step of the reaction, catalyzed by L-threonine aldolase, was also predicted to proceed in reverse in this strain design.

During genome-scale model reconstructions such as those conducted by Nogales et al., reversibility is determined from known biochemical data or estimated Gibbs free energy values, otherwise if no information is available it is set to be reversible. We recognize that setting too many reversible reaction when they are not may lead to potentially futile cycles, and it is important to recognize that this is another limitation of genome-scale modelling and FBA/FVA predictions since there is no thermodynamics (other than the assigned reaction reversibility) and reaction kinetics involved to be certain if one direction will be more thermodinamically favored than the other. Nevertheless, the L-threonine aldolase reaction is determined to be reversible in [MetaCyc](https://biocyc.org/META/NEW-IMAGE?object=THREONINE-ALDOLASE-RXN) and the original iJN1462 ([Nogales et al. 2020](https://ami-journals.onlinelibrary.wiley.com/doi/full/10.1111/1462-2920.14843)), which means it is allowed to proceed in either direction in genome-scale modelling.

***Please see Page-22, line-13.***

It is also important to note that FVA and flux-sum analyses are based on linear, static, constraint-based modeling and do not account for factors such as gene regulations, reaction kinetics, and cellular stress responses . . .

**Specific comment-1.3.11**

*“Although growth-coupling regimes exist for three of the tested aromatic compounds,…”, I do not see any values for ACT on the aromatics except for one. Could you please explain in detail?*

**Answer-1.3.11**

We thank the reviewer #1 for the valuable suggestions and comments. We have extended the sentence to remove ambiguity and clarify what we were referring to. That particular sentence was referring to the cMCS calculation on the six other aromatics, not the flux distribution analysis, which can be found in Supporting Information 3 under “other aromatic carbon sources”. We believe this was confused with the previous sentence, which was referring to Supporting Information 6, where we use the same strain designs for MR on the other aromatic carbon sources.

The analysis in Supporting Information 3 was performed to see whether the other aromatics could produce a growth-coupling regime based on cMCS, while the analysis in Supporting Information 6 is shown to confirm Banerjee et al’s observation that growth-coupling regimes found by cMCS on a substrate (MR) is not applicable to other substrates, because as reviewer #1 has mentioned, only 1 managed to produce ACT with the same strain design found on MR. Thus, if one is interested in finding growth-coupled designs on the other aromatics, they would have to implement the knockouts suggested in Supporting Information 3. However, this was not done in this study because we have focused our scope on using azo dyes for ACT production.

***Please see Page-35, line-5.***

We also expanded our cMCS search on several other aromatic carbons that utilize similar catabolic pathways as MR, namely benzoate, 4-hydroxybenzoate, vanillin, vanillate, ferulate, and gallate (Supporting Information 3). We found that growth-coupled regimes are only feasible on aromatics that have 0 net NADH or require at least a net of 1 NADH in their catabolism, namely benzoate, 4-hydroxybenzoate, and gallate. Growth-coupling was not possible on vannilate, vannilin, or ferulate. Moreover, we retrieved the flux distributions for the four suggested strain designs found on MR when grown on the six other aromatic substrates (Supporting Information 6, Table S1-S6). Although growth-coupling regimes exist for three of the tested aromatic compounds (Supporting Information 3), we found that the specific growth-coupled phenotype found on MR could not be replicated using the same strain design on other aromatics, further confirming that cMCS suggestions are unique to the carbon source specified prior to the cMCS computation.

**Specific comment-1.3.12**

*Does the growth coupling still hold when you optimize for growth in the strain designs?*

**Answer-1.3.12**

We thank the reviewer #1 for their valuable comments and questions. Yes, this is what we tested in Section 3.5. We believe this question has also been answered at length in Answer-1.3.9. Product yield, minimum guaranteed ACT yield, and BPCY are all calculated under growth as the objective.

**Specific comment-1.3.13**

*Fig 7a: Does the first blue bar for WT represent the theoretical max Act yield?*

**Answer-1.3.13**

We thank reviewer #1 for the questions and comments. Yes. This was the maximum possible ACT yield, i.e., the maximum yield calculated by FVA when ACT is the objective. Although we know from Section 3.2 that at maximum ACT, growth would be very low or none at all, this value shows the highest ACT yield the cell can theoretically achieve regardless of whether there is growth or not. In Fig. 7a, we see that WT can theoretically produce 0.12 ACT yield when biomass flux is 0 mmol/gDW/h, but no ACT is produced when biomass is optimized (hence no dark blue bar). Fig. 7a displays how much the strain designs can fulfill the theoretical ACT production potential.

**1.4 Conclusion**

**Specific comment-1.4.1**

*Considering you are advocating for MR from wastewater or dye industry effluents, how feasible, cost effective and sustainable do you think it is to supply this as a feedstock to a large-scale microbial fermentation process? Does the MR have to be separated first as other substrates in the wastewater might uncouple production.*

**Answer-1.4.1**

We thank reviewer #1 for the intriguing questions. As our study is heavily computational and relies on stoichiometric genome-scale modelling, at this point, we have limited evidence to make a well-informed prediction regarding the feasibility of the system in large-scale fermentation process. Generally, as long as the azoreductase is specific enough to allow *P. putida* to metabolize the present azo dyes only, other substrates in the wastewater should not fatally uncouple production, although this statement warrants further testing. However, given previous studies that show effective separation of azo dyes from wastewater effluents by electrocoagulation, adsorption, and reverse osmosis, we can expect that if these systems are combined with an engineered *P. putida* capable of azo dye catabolism, it would allow for a more eco-friendly degradation process that generates less unused byproducts and simultaneously produces industrially relevant compounds using the separated azo dye as substrate. A combination of chemical and microbial systems may be a more realistic possible implementation of this system. Furthermore, large-scale implementation of microbial systems is typically more cost-effective than chemical decolourization processes that require addition of reagents and specialized treatment plants.

We believe that the long-term benefits of implementing this system would overall outweigh the costs of building a *P. putida* strain capable of a growth-coupled azo dye to polyketide conversion.

We have revised the manuscript and included these points in the conclusions section.

***Please see Page-39, line-2.***

Industrial wastes such as azo dyes have the potential to be reused as feedstock for biomanufacturing high-value compounds and secondary metabolites. With the development of various azo dye separation processes from wastewater through adsorption, coagulation, and membrane filtration, azo dyes have become more realistically accessible for microbial systems to degrade and valorize into new compounds. Through this work, we have demonstrated that it is plausible to implement a system in *P. putida* KT2440 to produce ACT from MR.

**2^nd^ Round Revisions**

**General Comment:**

*The authors have written a well-thought-out response with necessary changes that considerably strengthen their hypothesis and enhance the flow of the manuscript. I do agree that this article is a nice demonstration of the effect of non-conventional carbon sources on target chemical production using genome scale models. Some of these nice rebuttal statements would actually make for a good conclusion or add-ons in the manuscript. Thanks to the authors for adding necessary references (for degradation of methyl red by P. putida) and the much improved and now legible Fig.4.*

**Response to reviewer #1:**

We thank reviewer 1 for the thoughtful suggestions and feedback that has helped improve the readability of the manuscript and strength of our arguments. We have evaluated additional comments raised by reviewer 1 and made revisions accordingly as detailed below.

**Specific comment-1**

*I still stand by my statement that an experimental demonstration would enhance the appeal of the work (and with a complete understanding of the limited access to funding and resources). By experimental demonstration - I do not mean the implementation of the time and labor-intensive growth coupling designs, rather the MR utilization by KT2440 as the sole carbon source is very vital to this study. The newly added citations are great examples for MR decolorization (maybe to a colorless product) but not utilization as a sole carbon source (those cells were grown on rich medium and MR already shows toxicity on rich medium). Going by the authors’ statements one might (worst case) or might not (best case) even need to express AnthDO to carry out this growth study. Therefore, I leave it to the editor’s discretion as to whether this is necessary for supporting the author’s claims. A recent and a relevant product substrate paired study using an aromatic compound from feedstocks (using p-coumarate as the sole carbon source) to Indigoidine by Eng et al., 2023 (Cell reports) in P. putida leaves room for discussion that even a 3 gene cut sets (plus only a 2-step heterologous pathway) needed lab evolution to improve growth even on a native aromatic carbon source.*

**Answer-1**

We appreciate the thoughtful comments raised by reviewer 1. We completely agree that an experimental demonstration of MR utilization by KT2440 would be a strong piece of evidence that supports the arguments presented in this paper. However, as pointed out by reviewer #1, to test if MR can be used as a *sole* carbon source, AnthDO, or at least the pWW0 TOL plasmid needs to be transformed into KT2440. If given the resources and opportunity to perform the experiment, I would have started by testing whether KT2440+TOL plasmid or *P. putida* mt-2 could grow with MR as a sole carbon source. This would test if the dioxygenases in the TOL plasmid are sufficient to metabolize anthranilate formed from MR reduction, otherwise, a heterologous AnthDO would need to be transformed as well. So far, there have been studies that tested growth of mt-2 in glucose media supplemented with azo dyes (Mansour et al.), but no study has attempted to grow them on azo dye only.

The newly added citation (Goncalves et al.) used *P. putida* MET94 (which has not been sequenced yet) and showed decolorization, not catabolism, of methyl red on rich media; and we do not know if MET94 possesses the TOL plasmid. However, based on the results of our study, we can hypothesize some possible explanations for the azo dye toxicity observed by both Mansour et al. and Goncalves et al:

1. As observed in the model, MR consumption inherently competes with growth above a certain flux due to the reaction requiring NADH, even in the presence of glucose. It is to be expected that in these studies, decrease in growth was observed as azo dye concentration was increased.
2. Decolorization occurs when MR/azo dye is converted into its aromatic amine. The cause of toxicity, however, are the aromatic amine byproducts. In the presence of glucose, glucose would be a preferred carbon source compared to anthranilate even though an anthranilate degradation pathway to tryptophan exists (see answer for comment #9). Therefore, in rich media, it is not surprising that some toxicity is still observed, because mt-2 (closest to the model), or MET94, could not consume anthranilate at a high enough flux via the existing tryptophan pathway. I would hypothesize that if one were to transform AnthDO, this likely would resolve or at least decrease toxicity, because it allows the cell to metabolize anthranilate at a higher rate (i.e., higher flux through AnthDO is possible compared to the tryptophan pathway based on the model). The fact that mt-2 containing the TOL plasmid still exhibited toxicity could be due to the presence of the preferred substrate glucose, or simply that the toluene dioxygenase was not able to convert anthranilate to catechol at a flux high enough to detoxify anthranilate (one needs to test growth without glucose before confirming the latter). Again, the fluxes predicted in the model represent the upper bound flux that could pass through this pathway and not the actual flux.

Finally, with regards to ALE, given more complicated interventions that need to be carried out for this study, it would be expected that ALE would need to be performed to optimize utilization of MR or other azo dyes in *P. putida* that is not well-adapted to these carbon sources.

**Specific comment-2**

*Since 2 of the 4 chosen designs talk about the meta-pathway for degradation and you mention KT2440 in the manuscript, this particular strain (not the in-silico model) is a TOL plasmid free strain (Nelson et al., 2002) and the genes for the meta-cleavage pathway for catechol degradation is located on the TOL plasmid. It would be prudent to suggest that any interested reader has to express these additional genes when they actually do the experiment. Also, as rightly pointed out by the authors, the ortho-cleavage pathway is highly regulated and although the model chooses the “shortest” or thermodynamically favorable path to the product according to the design interventions- it will not be the case in an actual cell where you might have to additionally delete the competing pathways for uptake/metabolism (the model has no deletion targets in these parallel pathways).*

**Answer-2**

We thank the reviewer for raising a very important point. As correctly mentioned by reviewer 1, the KT2440 strain is a cured strain and does not contain the TOL plasmid containing the meta cleavage pathway and several dioxygenases. Despite this, the original iJN1462 model still included the corresponding reactions to the reconstruction since the parental strain mt-2 contains the TOL pasmid and that it is used to expand the capabilities of KT2440 (Nogales et al. 2008). As suggested, we have revised the manuscript to mention the additional plasmid that would need to be expressed if readers were to perform the experiment with KT2440.

***Please see Page-16, line-16.***

It is important to note that although the iJN1462 model contains reactions encoded in the TOL plasmid, including the toluene-1,2-dioxygenase and the meta-cleavage pathway, *P. putida* KT2440 is a cured strain derived from *P. putida* mt-2 and does not possess the plasmid. For experimental demonstration of the designs presented in this study, one would have to express the TOL plasmid in KT2440 or use the mt-2 parental strain.

**Specific comment-3**

*Growth-coupling can actually be difficult to demonstrate for even routine secondary metabolites on different carbon sources. Thanks for the clarification on the choice of ACT. I do agree that it is a valuable and a suitable target (given its NADH production capability that seems to be the critical point to drive MR utilization and coupling with endogenous NADH generators knocked out). Whether it would pose a challenge to actually implement them (22 step ACT pathway +the many knockouts of the design + expression of the meta-cleavage pathway genes in KT2440) and discourage one from implementation (again for lack of funding/resources), I shall refrain from commenting at this stage. Could you perhaps add in a few more products in the discussion that are easier to implement (you need not validate but which might work using a similar mechanism?) if you find it suitable? This will be a food for thought to the readers and will motivate them to pursue this line of experiments.*

**Answer-3**

We thank reviewer 1 for their valuable suggestions. We have revised the manuscript and included other potential products that might be easier to implement than ACT in the conclusions.

***Please see Page-42, line-11.***

For example, one could potentially apply the same approach to growth-couple and produce acetate, acetoin, or 2,3-butanediol from MR which, although not a polyketide, also generate NADH in its biosynthesis similar to ACT.

**Specific comment-4**

*The model- is iJN1462 the latest model or 63? Also, could you please add in the reactions through the peripheral pathways (glucose to gluconate etc.) in Fig 5? What were the fluxes through them (apart from ED-EMP) on glucose +MR?*

**Answer-4**

We thank the reviewer for the question and suggestion. The model was listed as iJN1463 in the BiGG Model database, however, when we checked the relevant publication for this model, the most recent reconstruction is described in Nogales et al. (2020) and is designated as iJN1462 in that study.

In Figure 5, we only examined fluxes when grown on either MR or glucose (not mixed) because our goal was to observe where the carbon fluxes from MR and glucose are utilized, whether it is mainly channeled to ACT or biomass, and which pathways were active during growth in either substrate. If mixed substrates are used, FBA cannot distinguish whether the fluxes were channeled from glucose, or MR through the aromatic catabolic pathways (experimentally, this of course could be analyzed by 13C-labelling, but we cannot retrieve this information *in silico*).

We did not show fluxes at the gluconate node because both gluconate and 6-phosphogluconate are indicative of whether the gluconate shunt / ED pathway was activated. For the cases grown on GLC, all gluconate was converted to 6-phosphogluconate (fluxes consuming gluconate and producing 6-phosphogluconate was 1:1); and for cases grown on MR, the gluconate shunt / ED pathway was inactive.

**Specific comment-5**

*ATP maintenance reaction- Perhaps I was not clear enough. I was wondering if the reaction for ATP maintenance in the model was constrained to an experimental value or unconstrained?*

**Answer-5**

We appreciate the reviewer for clarifying the previous question. The ATP maintenance value in the model was constrained to 0.92 mmol/gDW/h as described in Nogales et al. (2020). This value is based on experimental data by Ebert et al. (2011), *Appl. Environ. Microbiol.* We did not alter the ATP maintenance value for our analyses.

**Specific comment-6**

*Section 2.5- From your response, I believe there was only one reaction “AnthDo” that was used for gap-filling? The description of the process in the methods section though seems like it went through a lot of iterations for a lot of reactions. Could you perhaps rephrase it?*

**Answer-6**

We thank reviewer 1 for their valuable feedback. Yes, during gapfilling, the addition of AnthDO was sufficient to enable MR conversion to ACT, thus it is the only reaction added in the process. We have rephrased the section as suggested and emphasized that AnthDO was the only reaction added to avoid misinterpretation of the methods.

***Please see Page-9, line-9.***

We used an FBA-based, model gap-filling method to construct our MR-to-ACT pathway since most of the metabolic framework is already native to *P. putida*. We started by defining the target metabolite, host, and growth medium, then ran FBA with ACTt as the objective and MR as the sole carbon source. If FBA returns an optimized value of the objective function, then a pathway to synthesize ACT from MR exists. The model is said to be unfeasible when no pathway was found to connect the two nodes. Initially, iJN1462b returned unfeasible on MR only and with ACTt as objective. To fill incomplete gaps in the pathway, we mapped the model in Escher to identify where the gaps lie and searched for compatible reactions in MetaCyc, NCBI GenBank, and UniProt to fill in missing links. If the model becomes feasible upon addition of a heterologous reaction, then that reaction is added to the model as part of the MR to ACT pathway. Otherwise, the process is repeated until it returns feasible. For our pathway, only one reaction, AnthDO, was needed to connect missing links in iJN1462b. The model containing the pathway that enabled MR conversion to ACT was designated iJN1462c (Supporting Information 1).

**Specific comment-7**

*Page-14 and line 10: Thanks for the clarification. Could you also confirm if the same applies to iJN1462a (without Act reactions as well)?*

**Answer-7**

We thank the reviewer for the question. Yes, iJN1462a without the ACT pathway also could not grow on MR alone even with AzoR_MR included. AnthDO is needed for the model to use MR as sole carbon source for growth or ACT production.

**Specific comment-8**

*Section 2.7: Although the authors might find it redundant or trivial, I would recommend that they at least make their codes (if not the details of each step) for everything available to readers for reproducibility. Apologies if it was included already in the supplementary.*

**Answer-8**

We thank reviewer 1 for their suggestions. We have revised our data availability section and made our codes available at https://github.com/parsanayyara/AzoDyePutida.

**Specific comment-9**

*On glc+MR medium or in a MR only medium (say in a fed-batch process), do you expect anthranilate to get converted to "trp" once serine becomes available through biosynthesis (not in silico but as a point of discussion for an actual scenario)? I feel that this alternate degradation pathway should be mentioned in the text as well if not already done. Your simulations in the response were pretty convincing and this fact could also advocate for a carbon specific growth coupling despite a competing pathway's presence.*

**Answer-9**

We thank reviewer #1 for providing thoughtful comments and interesting points of discussion. Yes, in an actual scenario, I would expect that once the reactants needed in the anthranilate-to-tryptophan pathway (i.e. serine or 5-Phospho-alpha-D-ribose-1-diphosphate (prpp[c])) becomes available through biosynthesis, then the cell would be able to convert anthranilate to tryptophan. This scenario could not be simulated through constraint-based modelling because constraint-based modelling is time-independent.

However, I have tested the cases in which both serine and MR/anthranilate were present, with the supplied serine’s flux lower than anthranilate’s flux (data available in GitHub repository). I expect that this scenario would be close to a point in time where low amounts of serine become available through biosynthesis. The test showed that although the cell can now grow on anthranilate and that it is converted to tryptophan, the flux through the anthranilate-to-trp pathway is still low in comparison to the overall biomass produced or serine supplied, indicating that it was serine that becomes the main source of carbon.

Similarly, with AnthDO on MR/anthranilate only, the flux through the tryptophan pathway is still very low compared to the flux through AnthDO. Therefore, it is likely that the anthranilate-to-tryptophan pathway was not used as the main route to catabolize anthranilate.

We have added a brief discussion about this alternate pathway as suggested in Section 3.1.

***Please see Page-16, line-22.***

*P. putida* also possess another pathway that converts anthranilate to tryptophan, which could be an alternative to the pathway via AnthDO. However, the conversion of anthranilate to tryptophan requires a supply of L-serine and 5-Phospho-alpha-D-ribose-1-diphosphate, which prevents this pathway from being the main route for anthranilate degradation. Without an external supply of either compounds or an additional carbon source to support biosynthesis, iJN1462b without AnthDO cannot grow or produce ACT from MR only (see supplementary notebook at the GitHub repository). Our supplementary tests also showed that although anthranilate is converted into tryptophan in the presence of an additional carbon source, the flux through the anthranilate-to-tryptophan pathway is low compared to the flux from the additional carbon source, indicating that anthranilate was not the main carbon source for growth. Similarly, in the presence of AnthDO with MR as sole carbon source, the flux through the tryptophan pathway is still very low compared to the flux through AnthDO. Therefore, despite the presence of an alternate anthranilate degradation pathway, AnthDO is still the preferred route to channel carbon flux from anthranilate to the central metabolic pathways.

**Reviewer: 2**

**Recommendation:** Publish after major revisions.

**General Comment:**

*The article employs flux balance analysis to showcase the promising potential of Azo dyes as an alternative feedstock compared to the commonly employed glucose-rich feedstocks in the production of polyketides. The production envelopes reveal that Methyl red (MR) surpasses glucose as a preferred feedstock for Actinorhodin (ACT) production. This preference is attributed to its lower demand for NADH flux in oxidative phosphorylation, resulting in reduced ATP turnover.*

*In the context of strain design, the authors looked for different gene knockouts to optimize the synthesis of ACT from MR, utilizing a constrained Minimal Cut Set (cMCS) approach for metabolic pathway analysis. This analysis indicates that the cMCS solutions are tailored to specific carbon sources.*

*In closing, this article delves into the potential of Azo dyes, with Methyl red as an alternative feedstock for polyketide production, particularly Actinorhodin. It could pave the way for non-sugar-based substrates to be used in the production of polyketides and other industrially important compounds.*

*While the research methodology is sound, the manuscript requires some essential refinements. The manuscript requires revision to include all the omitted information and rectify minor errors.*

**Response to reviewer #2:**

We thank reviewer #2 for their detailed comments and recommendations. As mentioned by reviewer #2, our results suggest that methyl red is predicted to be a better substrate choice than glucose for actinorhodin production as it allows growth-coupling regimes that are not possible with glucose due to its ability to create NADH demand and consumes excess NADH generated by actinorhodin synthesis. Suggestions provided by reviewer #2 have collectively helped strengthen our manuscript and clarify points that might be ambiguous or require further elaboration. We have carefully addressed each comment raised as follows.

**Specific comment-2.1**

*No hyperlinks or references are available for the updated metabolic model iJN1462c.*

**Answer-2.1**

We thank reviewer #2 for raising this concern. However, we have included the modified metabolic model iJN1462c in Supporting Information 1 in .xml format readable and modifiable in the COBRA and COBRApy environment. The iJN1462c, produced from this study, is essentially the iJN1462 model by Nogales et al. with the actinorhodin biosynthesis pathway and anthranilate-1,2-dioxygenase added for the purpose of our computation.

**Specific comment-2.2**

*Supporting information for the data used in the study is available upon request.*

**Answer-2.2**

We apologize for the confusion about the accessibility of our Supporting Information—all Supporting Information available and relevant has been included in the manuscript, there is no other Supporting Information that remains unattached.

**Specific comment-2.3**

*There’s inconsistency in the reported total number of MCS solutions for different biomass and ACT flux distributions, Page 22 line 1 mentions 203 solutions whereas line 3, mentions 213 MCS solutions.*

**Answer-2.3**

We thank the reviewer #2 for raising this concern. We have checked our manuscript and there were no inconsistencies in the paragraph. In page-23 line-1, we mentioned that there were 203 MCS solutions in M9 minimal media + MR, 10 solutions in M9 minimal media + GLC + MR, and 0 solutions in M9 minimal media with GLC as the sole carbon source. In page-23 line-3, we explained that “Out of the [total] 213 solutions, only 4 strain designs had a reasonable knockout size and a gene knockout phenotype that agrees with the reaction knockout phenotype suggested by cMCS”. The 213 MCS solutions were a total of all the solutions found in all media compositions.

**Specific comment-2.4**

*Pseudomonas putida should be italicized or underlined according to taxonomic nomenclature (Page 2, line 5) (Abstract).*

**Answer-2.4**

We thank reviewer #2 for this comment, and we have revised the manuscript as suggested.

**Specific comment-2.5**

*Table 1 would benefit from additional information about the reactions (Page 7).*

**Answer-2.5**

We thank reviewer #2 for this suggestion. We did not include additional information as we felt this was redundant given that most of these are transport and exchange reactions that are non-enzymatic, except for AzoR_MR. Additional information (i.e., reaction stoichiometry, directionality, any associated genes and enzymes) are also all retrievable from the model (Supporting Information 1) in COBRA. Nevertheless, we decided to revise the manuscript and include the reaction description in Table 1 for clarity.

**Specific comment-2.6**

*Prior to discussing iJN1462c, it's important to include information about iJN1462b (Page 7, line 5).*

**Answer-2.6**

We thank reviewer #2 for their recommendations. Information about iJN1462b has been included prior to the first mention of iJN1462c as shown below. We believe this provides all the information needed about iJN1462—iJN1462b is the iJN1462a model (which has the AzoR_MR reaction re-added to the model, previously discarded by Nogales et al.) with the actinorhodin biosynthetic pathway reactions from the *S. coelicolor* model included. Please accept that we have not decided to add further revisions on this matter.

***Please see Page-8, line-4.***

To simulate the engineered P. putida harboring the ACT gene cluster (iJN1462b), 22 essential reactions for ACT biosynthesis were added from iAA1259 into iJN1462a. iJN1462c (Supporting Information 1) was the model containing the complete heterologous pathway that enables azo dye conversion to ACT.

**Specific comment-2.7**

*To facilitate the calculation of the rate of cofactor regeneration using the flux sum concept, it would be helpful to include notations for various variables (Page 9, Equation 1).*

**Answer-2.7**

We thank reviewer #2 for this recommendation, we have revised the manuscript to include an explanation of the variables. Please see Page-10, Line-14, Equation 1.

**Specific comment-2.8**

*The source and calculation method for the MR uptake rate mentioned in Line 9 of Page 17 has not been provided.*

**Answer-2.8**

We thank reviewer #2 for this comment. In this study, the MR uptake rate was kept unbound prior to flux variability analysis as there are no previous studies to date that measure this experimentally in *P. putida* KT2440. Moreover, the rest of the model is well-constrained enough that the minimum and maximum MR uptake rate possible stoichiometrically can be calculated via flux variability analysis—this was what we did in our study. We also wanted to observe how much MR the cell can theoretically uptake with changing objectives and media compositions, which we tested through flux balance analysis and flux variability analysis. This is elaborated in detail in Section 2.4 as follows.

***Please see Page-9, line-4.***

Uptake rates for all nutrients were set according to the in silico M9 minimal medium as specified by Nogales et al., except for methyl red (MR), because its specific steady-state uptake rate in P. putida had not been tested experimentally before. Thus, because we aim to analyze the maximum theoretical capability of iJN1462c to consume MR in this study, the MR uptake rate in the medium, if any, was set to 1000 mmol gDW-1 h-1. The value 1000 was chosen to simulate an extreme case in which MR uptake was not constrained and the cell was free to consume as much MR as possible.

**Specific comment-2.9**

*The rationale for selecting only four design strains due to reasonable knockout size is missing; an interpretation of this should be included (Page 22)*

**Answer-2.9**

We thank reviewer #2 for this comment and suggestion. The criteria of selecting the strain designs to be analyzed further was 1) it has a knockout size of under 25 gene knockout targets and 2) the predicted phenotype produced by reaction knockouts suggested by cMCS agrees with the predicted phenotype produced by the simulated gene knockout (i.e., knocking out the genes corresponding to that reaction actually makes the reaction nonfunctional), the latter being more important. This point has been stated in page-12, line-14, Section 2.10 as follows. We have revised the manuscript and added a sentence to clarify our reasoning.

***Please see Page-12, line-18.***

To verify gene-protein-reaction (GPR) associations, we performed another knockout simulation using only gene knockouts and observed if the gene knockout agreed with reaction knockouts (Supporting Information 3). For reactions catalyzed by isoenzymes, genes encoding all known isoenzymes are knocked out. For reactions catalyzed by enzymes with subunits or encoded by multiple genes, only one gene is knocked-out since the associated enzyme would not be functional without one of the subunits. Knocking out genes controlling more than one reaction is avoided. Only strain designs where the gene knockout agrees with the suggested reaction knockouts are evaluated in the next step.

Additionally, the choice of 25 knockouts as a maximum is because more knockout means more genetic modifications in the cell. We have seen studies successfully using multiplex CRISPR knocking out up to 9 genes (e.g., Banerjee et al.) and up to 16 genes with RecET-based recombineering (Choi et al). More genetic manipulations are experimentally more exhaustive (given that for this hypothetical strain design we would also need to express the actinorhodin gene cluster) and even if those manipulations are successful, it is harder for the cell to maintain those modifications with the selective pressure from the wild-type strain.

**2^nd^ Round Revisions**

**General Comment:**

*The authors have revised the manuscript, incorporating the reviewers' suggestions and comments. The manuscript now presents a clear and comprehensive explanation, making it more accessible to a broader audience. The inclusion of metabolic maps and detailed tables with annotated equations further improves clarity.*

**Response to reviewer #2:**

We thank reviewer 2 for their suggestions that have helped improve the clarity of our manuscript. We have revised our manuscript in light of further comments provided by reviewer 2 as detailed below.

**Specific comment-1**

*To further highlight the novelty of your work, which considers different flux spaces with non-sugar-based sources, please provide examples and references for criteria of selecting carbon sources which does not take the solution space for the specific metabolite into account.*

**Answer-1**

We thank reviewer 2 for the thoughtful comment. We have included new references in Page 6 line-14 as examples of metabolic engineering efforts that considers either the host organism’s catabolic capabilities, environmental factors, or cost (or sometimes all three) when selecting carbon sources but have not evaluated the different solution sets that could be generated as a result. We should note that although these studies did not explicitly evaluate solution spaces when selecting carbon sources, it could still be the case that the carbon source chosen happen to be the one with the solution space most suitable for growth-coupling the target metabolite.

**Specific comment-2**

*Could you provide a brief explanation for focusing solely on the meta-cleavage pathway? Given that both meta and ortho cleavage pathways catabolize catechol to acetyl-CoA, a common entry point for the TCA cycle and fatty acid biosynthesis, this explanation would help readers better understand your decision-making process. (Page 16)*

**Answer-2**

We thank reviewer 2 for the questions and suggestions. We recognize that both meta and ortho cleavage pathways converge at the beta-ketoadipate node and thus can be used to convert catechol into acetyl-CoA. In the context of Page 16, we agree that we should have mentioned both the meta-cleavage and ortho-cleavage pathway when explaining the pathways involved in MR conversion to ACT because both pathways are indeed possible routes for the catabolism of aromatic compounds to acetyl-CoA; strain design 4 that was suggested by MCS utilizes the ortho pathway instead of meta.

We have revised the manuscript to mention the ortho-cleavage pathway and edited Figure 1 and 4 to highlight the ortho-cleavage pathway as an alternative possible route.

***Please see Page-17, line-13.***

Catechol produced via AnthDO is then catabolized into acetyl-CoA either via the meta-cleavage or ortho-cleavage pathway, connecting dye degradation to the central carbon metabolism.

**Specific comment-3**

*Would you be willing to provide a rationale or derivation for the threshold values of MR uptake rate? This will provide additional context and support for the derived values. (Page 19, line 3)*

**Answer-3**

We thank the reviewer for their feedback. There was no specific mathematical derivation done manually to determine the MR uptake rate thresholds. The “thresholds” were simply the maximum MR uptake rate the cell can theoretically handle before experiencing a decrease in growth rate as observed in the production envelopes in Fig. 2 A-D.

We believe this result and rationale has been sufficiently explained in Page 19 line-7; the fact that growth rate decreased when MR uptake rate values exceed a certain point (i.e., threshold) means that there is a competition between the fluxes of the two reactions. From the cofactor consumption and regeneration analysis (Supporting Information 2), we found that both processes needed NADH supply. Therefore, when we increase MR uptake rate above the “thresholds”, we increase the NADH consumed to catabolize MR, hence less NADH is available for respiration and growth.

To improve clarity, we have revised the manuscript and refrained from referring to this value as “threshold” and instead explained it as “the uptake rate attainable without decrease in growth or ACT”, to avoid confusion with the cMCS thresholds that was used in a separate section of the manuscript.

***Please see:***

***Page-20, line-3.***

Theoretically, the cell was able to consume MR up to 54.7 mmol gDW-1 h-1 with MR as the sole carbon source and 85.5 mmol gDW-1 h-1 with GLC+MR (Figure 2A and 2B); however, pushing MR consumption above **9.2 and 5.7 mmol gDW-1 h-1** would decrease cell growth.

***Page-20, line-15.***

Second, iJN1462c displayed an almost identical pattern of MR consumption when the objective was changed from BIOMASS_KT2440_WT3 to ACTt (Figure 2C and 2D), where MR uptake was also coupled with ACT production up until a specific **point**.

***Page-21, line-5.***

We predicted that the higher **MRt1 uptake rate attainable** by optimizing ACT compared to optimizing growth, might be due to differences in how cofactors were regenerated when the cell objective was changed.

***Page-22, line-1.***

Regardless of the objective, adding GLC into the medium lowers **the uptake rate at which MR can be consumed without any decrease in growth rate or ACT flux.**
